# An exploratory study examining how nano-liquid chromatography–mass spectrometry and phosphoproteomics can differentiate patients with advanced fibrosis and higher percentage collagen in non-alcoholic fatty liver disease

**DOI:** 10.1186/s12916-018-1136-1

**Published:** 2018-09-12

**Authors:** Zobair M. Younossi, Azza Karrar, Mariaelena Pierobon, Aybike Birerdinc, Maria Stepanova, Dinan Abdelatif, Zahra Younoszai, Thomas Jeffers, Sean Felix, Kianoush Jeiran, Alex Hodge, Weidong Zhou, Fanny Monge, Lakshmi Alaparthi, Vikas Chandhoke, Zachary D. Goodman, Emanuel F. Petricoin

**Affiliations:** 1 Betty and Guy Beatty Center for Integrated Research, Inova Health System, 3300 Gallows Rd., Falls Church, VA USA; 2 Department of Medicine, Inova Fairfax Hospital, Falls Church, VA USA; 3 Center for Liver Diseases, Inova Fairfax Hospital, Falls Church, VA USA; 4 Center for Applied Proteomics and Molecular Medicine, School of Systems Biology, George Mason University, Manassas, VA USA

**Keywords:** NASH, Steatosis, Steatohepatitis, Reverse phase protein arrays, Mass spectrometry, Liver fibrosis

## Abstract

**Background:**

Non-alcoholic steatohepatitis (NASH) is among the leading causes of liver disease worldwide. It is increasingly recognized that the phenotype of NASH may involve a number of different pathways, of which each could become important therapeutic targets. The aim of this study is to use high resolution mass spectrometry (MS) and phosphoproteomics techniques to assess the serum proteome and hepatic phosphoproteome in subjects with NASH-related fibrosis.

**Methods:**

Sixty-seven biopsy-proven NAFLD subjects with frozen sera and liver tissue were included. Reverse phase protein microarray was used to quantify the phosphorylation of key signaling proteins in liver and nano-liquid chromatography (LC)-MS was used to sequence target biomarkers in the serum. An image analysis algorithm was used to quantify the percentage of collagen (% collagen) using computer-assisted morphometry. Using multiple regression models, serum proteomes and phosphorylated hepatic proteins that were independently (*p* ≤ 0.05) associated with advanced fibrosis (stage ≥ 2) and higher % collagen were assessed.

**Results:**

Phosphorylated signaling pathways in the liver revealed that apoptosis signal-regulating kinase 1, mitogen-activated protein kinase (ASK1-MAPK pathway involving ASK1 S38 (*p* < 0.02) and p38 MAPK (*p* = 0.0002)) activated by the inflammatory cytokine interleukin (IL-10) (*p* < 0.001), were independently associated with higher % collagen. LC-MS data revealed that serum alpha-2 macroglobulin (α2M) (*p* = 0.0004) and coagulation factor V (*p* = 0.0127) were independently associated with higher % hepatic collagen.

**Conclusions:**

Simultaneous profiling of serum proteome and hepatic phosphoproteome reveals that the activation of ASK1 S38, p38 MAPK in the liver, and serum α2M and coagulation factor V are independently associated with hepatic collagen deposition in patients with NASH. These data suggest the role of these pathways in the pathogenesis of NASH-related fibrosis as a potential therapeutic target.

**Electronic supplementary material:**

The online version of this article (10.1186/s12916-018-1136-1) contains supplementary material, which is available to authorized users.

## Background

Non-alcoholic fatty liver disease (NAFLD) is rapidly becoming the most prominent cause of chronic liver disease worldwide, with prevalence estimated at approximately 24% [1]. NAFLD is considered the hepatic manifestation of metabolic syndrome and represents a spectrum ranging from steatosis to non-alcoholic steatohepatitis (NASH) and NASH-related cirrhosis [2]. Liver biopsy data suggest that approximately 20% of subjects with NAFLD have underlying NASH [3, 4]. Furthermore, NAFLD subjects with multiple components of metabolic syndrome have an increased likelihood of having underlying NASH and advanced fibrosis [3, 5, 6]. Evidence from observational studies suggests that NASH is the subtype of NAFLD with the highest risk of progression and adverse outcomes [2, 6]. However, a small proportion of subjects whose liver biopsies indicate non-NASH NAFLD may also progress to NASH and related fibrosis [7]. Nevertheless, the exact conditions under which some of these patients’ progress is still not well understood.

In addition to the clinical factors associated with adverse outcomes in NASH, a number of studies have assessed histologic features associated with mortality. In this context, the presence of histologic inflammation and ballooning degeneration of hepatocytes can be associated with advanced hepatic fibrosis in patients with NASH but not mortality [8–13]. In contrast, only hepatic fibrosis was shown to independently predict liver-related mortality [9, 12, 13]. This has prompted some experts to consider the presence of fibrosis in NASH as the most important predictor of long-term prognosis. It is important to note that, in 2017, histologic assessment remains the gold standard for diagnosing NASH and staging hepatic fibrosis [14–16]. Despite a great deal of ongoing efforts, a validated non-invasive biomarker or an effective treatment for NASH has not been approved [17–29].

Proteomics is a powerful technology that can help identify therapeutic targets and potential biomarkers in different diseases. We have utilized this high throughput technology in our previous studies of NASH in adipose tissue [21–24], where have shown that (1) NASH-specific disruption of the kinase-driven signaling cascades in visceral adipose tissue leads to detectable changes in the levels of soluble molecules released into the bloodstream, and (2) biomarkers discovered in silico could contribute to predictive models for chronic diseases.

An in-depth assessment of different proteomics platforms in patients with NASH and associated fibrosis is not available. Therefore, the present study aimed to use reverse phase protein microarray (RPMA) techniques to evaluate phosphoproteomic pathways that may be active in the hepatic tissue of patients with NASH-related fibrosis. Additionally, we aimed to use protein harvesting nanoparticles coupled with high resolution mass spectrometry (MS) to assess the serum proteome associated with NASH-related fibrosis.

## Methods

### Study design: study cohort, blood and liver tissue collection

The study cohort included 67 obese patients (BMI > 40 kg/m^2^) who underwent a liver biopsy for clinical indications. Histological diagnosis was made by the study hepatopathologist (ZG) and patients were classified into groups as NASH NAFLD (*n* = 42) and non-NASH NAFLD (simple steatosis) (*n* = 24) or patients with fibrosis stage ≥ 2 (*n* = 5) and fibrosis stage 2 < (*n* = 3). Fibrosis was also quantified after staining for collagen type V by Sirius red staining as outlined in detail below .

Blood samples, liver tissue, and demographic and clinical data were collected at the time of liver biopsy and after obtaining informed consent. All human samples were collected according to a protocol approved by Inova Fairfax Hospital Institutional Review Board and under patient informed written consent. Consistent with the intent of the study, patients diagnosed with viral hepatitis (HCV antibody (−) and HBs Antigen (−)) or other liver diseases, hepatocellular carcinoma, and/or evidence of excessive alcohol use (≥ 10 g/d), as defined by established clinical criteria, were excluded.

### Liver histological assessment and collagen quantification

All liver biopsies were stained with hematoxylin and eosin and Sirius red for steatosis and collagen quantification. All biopsies were evaluated by the study hepatopathologist for the degree of steatosis (0–3), portal inflammation (0–3), interlobular pericellular fibrosis, portal fibrosis (0–3), and the presence of bridging fibrosis and cirrhosis. The definition of NAFLD included presence of more than 5% macrovesicular hepatic steatosis. NASH diagnosis required presence of hepatic steatosis and inflammation, together with hepatocyte injury with degenerative ballooning, with or without Mallory–Denk bodies and/or pericellular fibrosis [30, 31]. Advanced fibrosis was defined as stage ≥ 2. Collagen quantification was performed following the acquisition of digitalized images of Sirius stained slides with an Aperio Scanscope XT scanner. Aperio’s positive pixel count algorithm was used to quantify the percentage of stained collagen (% collagen) (Additional file 1: Figure S1). Values for % collagen were grouped into upper quartile (Q4 > 5.36% *n* = 15), middle quartiles (Q2–Q3 = 1.58–5.36, *n* = 31), and lower quartile (Q1 < 1.89%, *n* = 16).

### Proteomics analysis

#### Serum proteome enrichment using nanoparticles

Hydrogel nanotrap particles were used to enrich for low abundance serum proteins as previously described [32]. Breifly, particles were prepared with three different affinity dyes (Trypan blue, Cibacron blue, and Bismark brown) that have been previously shown to be an optimal recipe to capture a wide range of low abundance proteins [33, 34]. Proteins were then released from the particles by elution with acetonitrile and ammonium hydroxide, followed by centrifugation.

#### Serum proteomics using liquid chromatography (LC) coupled with tandem MS analysis

Prior to MS analysis, nanoparticle-captured eluates were dried under compressed nitrogen and subsequently reconstituted in 8 M urea, reduced by 10 mM DTT, alkylated by 50 mM iodoacetamide, and digested by trypsin at 37 °C overnight. Tryptic peptides were further purified by ZipTip (Millipore, Billerica, MA, USA) and analyzed by LC coupled tandem MS (LC–MS/MS) with an LTQ-Orbitrap mass spectrometer (Thermo Fisher Scientific, Waltham, MA, USA). The reversed-phase LC column was packed with resin (Michrom BioResources, CA, USA) and then washed with 0.1% formic acid after sample injection and peptides were eluted using a linear gradient of 0% mobile phase B to 50% B to 100% B. The LTQ-Orbitrap was operated in a data-dependent mode in which each full MS scan (30,000 resolving power) was followed by eight MS/MS scans where the eight most abundant molecular ions were selected and fragmented by collision-induced dissociation using a normalized collision energy of 35%. The ‘FT master scan preview mode’, ‘Charge state screening’, ‘Monoisotopic precursor selection’, and ‘Charge state rejection’ were permitted so that only 1+, 2+, and 3+ ions were selected and fragmented by collision-induced dissociation. Tandem mass spectra data collected by Xcalibur (version 2.0.2) were examined in the NCBI human protein database using SEQUEST (Bioworks software, ThermoFisher, version 3.3.1). The SEQUEST blast search was further filtered by the criteria ‘Xcorr versus charge 1.9, 2.2, 3.0 for 1+, 2+, 3+ ions; ΔCn>0.1; probability of randomized identification of peptide < 0.01’. The false discovery rate of peptides was evaluated by searching a combined forward-reversed database. The search result files from each sample were further analyzed by Scaffold (Proteome Software, Portland, OR, USA) for further comparison [34, 35]. Only proteins that were detected in at least 50% of samples were included in the analysis.

#### RPMA construction and analysis performed on the hepatic phosphoproteins

RPMA was performed on the liver tissue to map pathway activation as previously described [22, 24]. Whole tissue lysates were prepared directly from 8 μm cryosections using a solution of 2× tris-glycine SDS sample buffer (Invitrogen Life Technologies, Carlsbad, CA, USA) and Tissue Protein Extraction Reagent (Pierce, Rockford, IL, USA) supplemented with 2.5% β-mercaptoethanol (Sigma, St. Louis, MO, USA). Samples were boiled for 8 min and stored at − 80 °C until arrayed.

Cell lysates were printed onto nitrocellulose-coated glass slides (Grace Bio-Labs, Bend, OR, USA) using an automated arrayer system (Aushon 2470 arrayer; Aushon BioSystems, Burlington, MA, USA). Each sample was printed in triplicate alongside the reference standards as an internal quality control. To quantify the overall amount of protein in each sample, selected arrays were probed with Sypro Ruby Protein Blot Stain (Molecular Probes, Eugene, OR, USA). Prior to immunostaining, samples were incubated in Reblot Antibody Stripping solution (Chemicon, Temecula, CA, USA), washed twice in PBS, and blocked in I-Block (Applied BioSystems, Foster City, CA, USA); 150 antibodies were used to target phosphorylated, cleaved, and unmodified proteins. Detection was performed using tyramide-based Catalyzed Signal Amplification System (Dako Cytomation, Carpinteria, CA, USA) coupled with the Streptavidin-conjugated IRDye680 dye (LI-COR Biosciences, Lincoln NE, USA). Antibody specificity was validated for single band specificity by western blotting prior to use on the arrays, as described previously [36]. Antibody- and Sypro Ruby-stained slides were scanned on a Tecan laser scanner (Tecan, Mönnedorf, Switzerland) using 620 nm and 580 nm weight length channels, respectively. Array images were analyzed using MicroVigene software version 5.1.0.0, as previously described [37–40]. Briefly, the software calculates spot intensity, completes background subtraction, normalizes each sample to its matched amount of total protein, and averages the technical replicates (Vigenetech, Carlisle, MA, USA).

### Statistical analysis

The clinical, demographic, and biochemical data of the study cohort groups were summarized as frequency (n), percentages (%), and mean ± standard deviation (SD). Mann–Whitney U test was used for continuous variables and χ^2^ was used to compare categorical data across disease states (NASH NAFLD vs. non-NASH NAFLD or advanced fibrosis, fibrosis stage 2 ≥ vs. fibrosis stage 0–1). In the context of multiple comparisons, false discovery rates were estimated using the Benjamini–Hochberg method. Spearman’s Rho (ρ) correlation analysis was performed across the detected analytes measured by MS and RPMA for patients with advanced fibrosis and higher % collagen. Correlation maps were created in Gephi version 0.8.2 for association with a correlation coefficient greater than 0.9.

Multiple regressions were built with analytes being used as predictors of the presence of significant fibrosis (logistic regression) and % collagen in liver (generalized linear model) with adjustment for demographic, clinical, and biochemical confounders (age, sex, BMI, diabetes, AST, and ALT). To limit the chance of over-fitting, the analytes were preselected at the univariate stage (*p* < 0.10 by Mann–Whitney or Spearman correlation test), and only predictors with *p* < 0.05 were left in the models after bidirectional stepwise selection. All statistical analyses were performed using statistical software JMP 9.2 (SAS Institute, Cary, NC, USA).

The open access mapping tool KEGG Pathway Painter, which uses The Kyoto Encyclopedia of Genes and Genomes (KEGG) database, was used to map the phosphoproteins determined from the multiple regression models to canonical pathways. The pathways were sorted in descending order based on number of molecules per pathway [41]. UniProt was used to manually curate the pathways and regulatory mechanisms involving the analytes found to be statistically significant in this analysis (http://www.uniprot.org/).

## Results

### Clinical and demographic profile of the study cohort

A total of 43 subjects with available liver tissue and serum samples met the histologic criteria for NASH and, of these, 34 had advanced fibrosis (fibrosis ≥ 2). Demographic, clinical, and histological data are summarized in Table 1, which shows the characteristics of the study cohort when divided as having NASH versus non-NASH. The *p* value column shows results of the χ^2^ test for categorical variables and Mann–Whitney U test for continuous variables, where *p* values < 0.05 are considered significant.

**Table 1:** Demographic, clinical, and biochemical characteristics of the study cohort

N	NASH	Non-NASH	All
	43	24	67
Demographic			
Male, *n* (%)	16 (37.2%)	6 (25.0%)	22 (32.8%)
White, *n* (%)	31 (77.5%)	19 (82.6%)	50 (79.4%)
Age mean ± SD	47.233 ± 10.254	45.083 ± 10.746	46.463 ± 10.403
BMI, kg/m^2^	48.921 ± 10.552	48.745 ± 9.200	48.856 ± 10.001
Clinical and biochemical			
Diabetes, *n* (%)	28 (65.1%)	8 (33.3%)*	36 (53.7%)
Hypertension, *n* (%)	30 (73.2%)	14 (58.3%)	44 (67.7%)
ALT, μ/L	51.214 ± 34.985	34.042 ± 18.320*	44.970 ± 30.985
AST, μ/L	40.619 ± 24.883	23.375 ± 12.968***	34.348 ± 22.802
Bilirubin, mg/dL	0.544 ± 0.040	0.383 ± 0.034**	0.486 ± 0.030
Albumin, g/dL	4.012 ± 0.114	4.127 ± 0.067	4.052 ± 0.078
Glucose, mg/dL	125.268 ± 6.310	111.130 ± 8.946	120.188 ± 5.193
Total cholesterol, mg/dL	175.025 ± 6.628	197.000 ± 8.732	183.266 ± 5.407
LDL, mg/dL	97.811 ± 6.199	120.043 ± 8.859	106.333 ± 5.259
HDL, mg/dL	40.667 ± 1.639	47.208 ± 2.438**	43.159 ± 1.422
TGA, mg/dL	172.103 ± 13.111	161.391 ± 23.062	168.129 ± 11.788
Histological features^a^			
Fat (1,2) vs. (3,4)	14 (32.6%)	5 (20.8%)	19 (28.4%)
Inflammation			
Portal inflammation (0.1) vs. (≥ 2)	15 (34.9%)	6 (25.0%)	21 (31.3%)
Kupffer cells (0.1) vs. (≥ 2)	11 (25.6%)	1 (4.2%)*	12 (17.9%)
PMN cells (0.1) vs. (≥ 2)	5 (11.6%)	0 (0.0%)	5 (7.5%)
Lymphocytes (0.1) vs. (≥ 2)	26 (60.5%)	6 (25.0%)**	32 (47.8%)
Fibrosis			
Pericellular fibrosis (0.1) vs. (≥ 2)	28 (65.1%)	0 (0.0%)***	28 (41.8%)
Portal fibrosis (0.1) vs. (≥ 2)	28 (65.1%)	7 (29.2%)**	35 (52.2%)
Bridging fibrosis and cirrhosis	14 (32.6%)	0 (0.0%)***	14 (20.9%)
Apoptosis			
Focal necrosis (0) vs. (1,2,3)	20 (46.5%)	5 (20.8%)*	25 (37.3%)
Mallory–Denk bodies (0) vs. (1,2,3)	12 (27.9%)	0 (0.0%)**	12 (17.9%)
Ballooning degeneration (0) vs. (1,2,3)	24 (55.8%)	0 (0.0%)***	24 (35.8%)
Apoptotic bodies (0) vs. (1,2,3)	4 (9.3%)	0 (0.0%)	4 (6.0%)
Collagen percentage	4.708 ± 3.283	2.794 ± 1.736*	4.040 ± 2.970
Fat percentage	18.645 ± 8.474	12.906 ± 7.552**	16.641 ± 8.559

^a^ Histopathological features were grouped by scores according to none, mild to moderate, or severe according to the hepatopathologist scoring as shown beside each variable

*p < 0.05, **p < 0.01, ***p < 0.001

*ALT* alanine aminotransferase, *AST* aspartate aminotransferase, *BMI* body mass index, *HDL* high density lipoprotein, *LDL* low density lipoprotein, *NASH* non-alcoholic steatohepatitis, *PMN* polymorphonuclear cells, *SD* standard deviation, *TGA* triglycerides

### Phosphorylated proteins in the hepatic tissue associated with advanced fibrosis and higher hepatic collagen deposition in subjects with NAFLD

The hepatic phosphoproteomic data was used for pathway activation mapping analysis. The analysis showed that 75 proteins (receptor tyrosine kinases, upstream activators, and downstream substrates) were associated with significant hepatic fibrosis (fibrosis stage ≥ 2) and higher % hepatic collagen. All significant RPMA proteomes in the liver are shown in Additional file 1: Table S1, whereas Table 2 shows the top 34 significant proteins. The false discovery rate was estimated to be 10.6%.

**Table 2:** Phosphorylated hepatic proteins with correlations with fibrosis and a higher percentage of collagen deposition in livers of NAFLD patients

Endpoint	Protein intensity values (mean)		
MITOGENESIS	Fibrosis (stage ≥ 2)^a,b^	Fibrosis (stage < 2)^a,b,c^	Percentage collagen ρ^c,d^
RTKS and LIGANDS			
c-Kit Y703	12,023.3253	8509.7594**	0.2727*
EGFR Y1045	7752.7248	5367.3954**	0.3061*
ErbB2 HER2 Y1248	13,638.4722	10,163.0936**	0.2544*
Met Y1234/1235	8394.0971	5442.5366***	0.4082***
Ret Y905	4840.5597	2796.3384**	0.2564*
Ron Y1353	11,455.9458	8217.8116**	0.2564*
DOWNSTREAM SUBSTRATES			
CrkII Y221	11,296.7507	7283.2336**	0.2844*
c-Myc	16,583.2182	13,624.0484*	0.2705*
ERK 1/2 TOTAL	27,683.0657	22,584.3185**	0.3574**
FRS2 alpha Y436	6745.9092	4569.8235*	0.3382**
IRS 1 S612	6603.2819	4223.5915*	0.2756*
PKA C T197	13,849.3962	11,729.8932*	0.2968*
Src Family Y416	3488.0194	1874.4209**	0.2631*
SURVIVAL			
AKT T308	19,966.2896	11,089.2265**	0.3763**
AKT TOTAL	15,739.0600	10,127.1820**	0.3282**
ASK1 S83	2072.4277	1209.4175**	0.2563*
Ephrin A3 Y779/A4 Y779/A5 Y833	10,051.2853	9008.6468*	0.3179*
PDK1 S241	13,526.9204	10,692.6456**	0.2990*
PI3K p85 Y458 p55 Y199	5930.4957	4023.6630**	0.4243***
PTEN	26,544.6698	22,904.4300*	0.2628*
PTEN S380	12,498.2321	7720.4331***	0.2677
INFLAMMATION/IMMUNE FUNCTION			
Zap 70 Y319/Syk Y352	8322.9034	4839.8932***	0.3189*
cPLA2 S505	11,697.1946	8108.4437**	0.2861*
PLCgamma1 Y783	11,995.7606	8488.3315**	0.2824*
Stat4 Y693	8407.7191	5859.6088**	0.3010*
Stat6 Y641	7840.7122	4793.4883***	0.3226*
Tyk2 Y1054/1055	8760.8030	5060.9800***	0.2955*
p38 MAPK T180/Y182	8093.9200	6807.1800	0.3901**
APOPTOSIS			
Bak	20,974.0624	18,147.0249**	0.3262**
Survivin	11,593.7421	8882.5924***	0.3180*
MOTILITY AND CELL ADHESION			
Cofilin S3	11,887.6509	2171.5929***	0.3369**
FAK Y576/577	7300.4566	5303.6335*	0.3382**
OTHERS			
ALDH	21,453.9429	26,006.4687**	−0.3009*
IL-10	1731.1300	1311.5200	0.5539***

^a^ Proteins in liver tissue, detected using RPMA, that were expressed at higher and significant level in patients with fibrosis ≥ 2 compared with fibrosis < 2

^b^ Non-parametric Kruskal–Wallis H test was used to identify the significantly differentially expressed proteins between the two groups

^c^ Protein level correlation with % collagen tested using Spearman rank correlation test

^d^ Correlation coefficient (ρ) describes the strength and direction of association between proteins and % collagen

*p* values < 0.05 are considered significant; *p < 0.05, **p < 0.01, ***p < 0.001

The phosphorylated signaling pathways from the liver tissue included the phosphoinositide 3-kinase PI3K/AKT signaling pathway, the epidermal growth factor receptor (EGF/EGFR) signaling pathway known as (ErbB1/HER1) and, most importantly, the Apoptosis Signal-Regulating Kinase 1 (ASK1)-MAPK pathways (involving Apoptosis Signal-Regulating Kinase-1, ASK1 S83, and p38 MAPKinase; *p* < 0.05). All phosphoproteins that were significantly correlated with the presence of advanced fibrosis and higher % collagen deposition in the liver are shown in Table 2.

As depicted in the protein–protein interaction figure (Additional file 1: Figure S2), the majority of phosphoproteins relevant to advanced fibrosis in NASH are linked together to form a large interacting network. This protein–protein interaction network revealed pathways involved in the neoangiogenesis, cell motility, and immune response to be potentially playing a role in the development of advanced fibrosis and higher % collagen deposition in NASH. Furthermore, these results suggest that the proteins of fibrosis and collagen deposition network have an even more pronounced hierarchical structure than the complete network and contain a larger number of closely linked protein clusters, which could be indicative of functional protein complexes.

### Serum protein profiles of LC–MS of NASH subjects with advanced fibrosis and increased hepatic collagen deposition

Important circulating proteins in sera (proteins that were detected by MS in at least 50% of NAFLD patients) were included in the further analysis. In the subsequent analysis, the strongest associations with advanced fibrosis were noted for the serum alpha-1-microglobulin/bikunin preprotein (*p* = 0.0022), the alpha-2 macroglobulin precursor (α2M) (*p* = 0.0023), and apolipoprotein E isoform a (*p* = 0.0027) (Table 3).

**Table 3:** Correlations of LC-MS serum proteomes with advanced fibrosis and a higher percentage of collagen deposition in liver

LC–MS Proteomes	Mean of protein intensity		
	Fibrosis ≥ 2 (n)^a,b^	Fibrosis < 2 (n)^a,b,c^	Percentage collagen ρ^c,d^
Protein alpha-1-microglobulin/bikunin preproprotein	0.170 ± 0.101	0.233 ± 0.091**	−0.25*
Alpha-2-macroglobulin	0.144 ± 0.144	0.060 ± 0.087**	0.32*
Apolipoprotein E isoform a	0.320 ± 0.184	0.426 ± 0.219**	−0.24
Transthyretin precursor	0.084 ± 0.056	0.053 ± 0.032**	0.26*
Vitamin K-dependent protein S preproprotein	0.057 ± 0.044	0.031 ± 0.030*	−0.06
Selenoprotein P isoform 2	0.013 ± 0.019	0.030 ± 0.044*	−0.19
Carboxypeptidase B2 isoform 2 preproprotein	0.025 ± 0.030	0.045 ± 0.042*	−0.24
Apolipoprotein C-II	0.115 ± 0.038	0.156 ± 0.093*	−0.19
Histidine-rich glycoprotein isoform X1	0.497 ± 0.252	0.619 ± 0.230*	−0.31*
Apolipoprotein B-100 isoform X1	0.250 ± 0.606	0.039 ± 0.073*	0.23
Apolipoprotein A-I preproprotein	0.753 ± 0.372	0.582 ± 0.222*	0.16
Inter-alpha trypsin inhib. hvy. ch.H4 isoform 2	0.045 ± 0.040	0.027 ± 0.030*	0.05
Coagulation factor V	0.048 ± 0.053	0.046 ±0.053	−0.36**
Alpha-1-antitrypsin	0.193 ± 0.192	0.121 ± 0.051*	0.34**
Platelet basic protein preproprotein	0.081 ± 0.053	0.100 ± 0.044	−0.33**
Vitamin D-binding protein isoform 3	0.052 ± 0.064	0.028 ± 0.037	0.28*
C4b-binding protein alpha chain	0.107 ± 0.084	0.102 ± 0.086	−0.27*
Complement C4-A isoform 1 preproprotein	0.589 ± 0.269	0.605 ± 0.212	− 0.26*
Alpha-1-acid glycoprotein 1 precursor	0.090 ± 0.062	0.065 ± 0.041	0.25*

^a^ Proteins as detected by LC–MS that were expressed at higher and significant level in patients with fibrosis ≥ 2 compared with fibrosis < 2

^b^ Non-parametric Kruskal–Wallis H test was used to identify the significantly differentially expressed proteins between the two groups

^c^ Protein level correlation with % collagen tested using Spearman rank correlation test and the

^d^ Correlation coefficient (ρ) describes the strength and direction of association between proteins and % collagen

*p* values < 0.05 are considered significant; *p < 0.05, **p < 0.01

On the other hand, α2M precursor coagulation factor V precursor and transthyretin precursor were weakly but significantly correlated with % collagen (*p* < 0.05) (Table 3). Furthermore, acute phase protein alpha-1-acid glycoprotein 1 was noted to positively correlate with % collagen while the platelet basic protein preproprotein (a chemoattractant which also functions in hyaluronic acid synthesis), histidine-rich glycoprotein isoform X1 coagulation factor V precursor, and alpha-1-microglobulin/bikunin preproprotein were all negatively correlated with % hepatic collagen deposition (*p* < 0.05) (Table 3). The false discovery rate for this round of analysis was estimated to be 28.3%.

#### Serum proteome and hepatic phosphoproteome independently associated with advanced hepatic fibrosis and increased hepatic collagen deposition in NASH

In a series of multivariate analyses, we assessed the independent association of these circulating serum proteins and hepatic phosphoproteins with advanced fibrosis and higher % hepatic collagen deposition (Table 4). Our initial models focused on advanced fibrosis as the outcome. The first multivariate model included hepatic phosphoproteins and suggested that phosphoprotein Tyk2 as well as ALDH were the independent predictors of advanced fibrosis in NASH (Table 4). Another multivariate model using serum proteins suggested that serum Apolipoprotein C-II precursor, Apolipoprotein A-I preproprotein, and vitamin K-dependent protein S preproprotein were independently associated with advanced fibrosis in NASH (Table 4).

**Table 4:** Multivariate models showing independent association of sera proteomes and phosphorylated protein in liver associated with advanced liver fibrosis and a higher percentage of collagen

Independent predictors of advanced liver fibrosis			
Predictor	Odds ratio	Lower 95% CI	Upper 95% CI
Model with RPMA proteins^a^			
AST, per 1 U/L	1.037	1.006	1.07*
Tyk2 Y1054/1055, per 1 unit	1.458	1.177	1.807***
ALDH, per 1 unit	0.873	0.783	0.973*
Model with MS proteins			
Apolipoprotein C-II precursor, per 1 unit	0.757	0.612	0.936*
Apolipoprotein A-I preproprotein, per 1 unit	1.086	1.036	1.138***
Vitamin K-dependent protein S preproprotein, per 1 unit	1.591	1.221	2.071***
Independent predictors of higher percentage collagen			
	Beta		Std. err.
Model with RPMA proteins^a^			
ASK1 S83, per 1 unit	− 0.654		0.280*
Met Y1234/1235, per 1 unit	0.450		0.167**
p38 MAPK T180/Y182, per 1 unit	0.290		0.072***
IL-10, per 1 unit	1.197		0.255***
LIMK1 T508/LIMK2 T505, per 1 unit	− 0.386		0.130**
Model with MS proteins			
Alpha-2-macroglobulin precursor, per 1 unit	0.100		0.026***
Coagulation factor V precursor, per 1 unit	− 0.159		0.062*

^a^ Liver tissue RPMA proteins were divided by 1000, MS proteins multiplied by 100 for presentation purposes

*p < 0.05, **p < 0.01, ***p < 0.001

The subsequent models focused on % collagen as the desired outcome. The model assessing the association of serum proteins with % collagen revealed that α2M precursor (*p* = 0.0004) and coagulation factor V (*p* = 0.013) were independently associated with increased hepatic collagen.

The last model showed that phosphoproteins, ASK1 S38 (*p* = 0.02), the receptor tyrosine kinase, Met Y1234/1235 (*p* = 0.009), p38MAPK T180/Y182 (*p* = 0.0002), LIMK1T508/LIMK2 T505 (*p* = 0.004), and tissue remodeling-related inflammatory cytokines IL-10 (*p* < 0.0001) were independently associated with a higher % collagen deposition in the liver (Table 4).

The complete list of parameters for the models also included age, gender, BMI, diabetes, AST, ALT, and the variables were then subjected to stepwise selection. The proteins included in the models (for further stepwise selection) were pre-selected at the univariate step (*p* ≤ 0.10 only). In the final models, only significant associations were kept for both clinicals and proteins (*p* ≤ 0.05).

#### Pathway analysis associated with increased hepatic collagen deposition using KEGG and Uniport

Based on the independent predictors of advanced fibrosis in NASH, the total number of pathways activated by phosphoprotein predictors is 122 (Additional file 1: Figure S3). Pathways of advanced fibrosis in NASH showed that there was an overlap of the Apolipoprotein C-II precursor, Apolipoprotein A-I preproprotein in the HDL-mediated lipid transport, and the Retinoid metabolism and transport pathways. On the other hand, Tyk2 is mapped to a total of 11 pathways, including Th1, Th2, and Th17 cell differentiation pathways (Additional file 1: Table S2).

Based on the independent predictors for increased % collagen deposition, pathway analysis revealed 173 distinct KEGG and UniProt pathways. Of these, 117 pathways involved the phosphoproteins (ASK1, Met, p38 MAPK, IL-10, LIMK1/LIMK2), 30 of which had two or more proteins per pathway. Met and p38 MAPK both mapped to the Adherens junction pathway (*n* = 2), both upstream of actin polymerization. ASK1 mapped to a total of 11 pathways, including Apoptosis, TNF, and NAFLD signaling pathways. LIMK1/LIMK2 mapped to 3 pathways, the most notable of which is the regulation of actin cytoskeleton pathway, which also includes p38MAPK. Pathways with the greatest number of convergent phosphoproteins included the Regulation of Actin Cytoskeleton (*n* = 3), Fc gamma R-mediated phagocytosis (*n* = 3), TNF signaling (*n* = 2), MAPK signaling (*n* = 2), Apoptosis (*n* = 2), and EGFR tyrosine kinase inhibitor resistance (*n* = 2). Additionally, IL-10 mapped to 20 pathways and was the only protein mapping to the canonical Jak-STAT signaling pathway (*n* = 1), solidifying its role as an upstream signaling molecule (Additional file 1: Figure S3 and Table S3).

Serum analytes, α2M, and Coagulation Factor V precursor were found to be involved in a total of 25 molecular functions and biological pathways (Additional file 1: Table S2) using Uniprot manual curation. Most notably, aside from their involvement in lipid and vesicle transport, both of these analytes mapped to Platelet degranulation and Fibrin Clot Formation pathways (http://www.uniprot.org/uniprot/P12259 and http://www.uniprot.org/uniprot/P01023).

### Correlations of MS proteins with independent predictors of fibrosis and higher % collagen

To investigate the relationships between the sera-derived proteins (detected by LC–MS) and tissue-derived phosphoproteins (assessed by RPMA) in the context of NASH-related fibrosis and hepatic collagen deposition, a separate analysis was performed. This analysis showed that the α2M precursor from serum was strongly and positively correlated with the receptor tyrosine kinase Met Y1234/1235 (ρ = 0.42, *p* = 0.0005), as well as with the actin-binding kinase LMK1 T508/LIMK2 T505 (ρ = 0.39, *p* = 0.001) and, most importantly, with IL-10 (ρ = 0.28, *p* = 0.02) and ASK1 S83 (ρ = 0.25, *p* = 0.04) from liver tissue samples (Additional file 1: Figure S4). Further correlations between proteins from sera and phosphorylated proteins from liver tissue can be seen in Table 5, which shows the correlation of the independently associated serum proteins (with fibrosis and % collagen) detected by MS with the independently correlated phosphoproteins detected by RPMA (with fibrosis and % collagen) in the liver. Spearman rank correlation test was used and correlation coefficient (ρ) describes the strength and direction of association between serum and tissue proteins.

**Table 5:** Correlations between hepatic phosphoproteins and serum proteins in NASH with advanced fibrosis and a higher percentage of hepatic collagen deposition in the liver

Serum proteome by LC-MS	Alpha-2-macroglobulin precursor	Coagulation factor V precursor	Apolipoprotein A-I preproprotein	Apolipoprotein C-II precursor	Vitamin K-dependent protein S preproprotein
Phosphoproteins from RPMA	ρ	ρ	ρ	ρ	ρ
ASK1 S83	0.25132*	− 0.27301*	0.25187*	0.18546	− 0.004
Met Y1234/1235	0.42158***	− 0.32161**	0.3759**	0.08211	0.2009
p38 MAPK T180/Y182	0.23536*	− 0.37073**	0.814	0.22551	− 0.1066
IL-10	0.27903*	− 0.38985***	0.2616*	0.11275	− 0.859
LIMK1 T508/ LIMK2 T505	0.39353***	− 0.3773	0.3206**	0.33034**	− 0.2423*
Tyk2 Y1054/1055	0.41652***	− 0.167	0.18053	0.01864	0.0099
ALDH	− 0.07782	− 0.004	− 0.275	0.109	− 0.020

*p* values < 0.05 are considered significant; *p < 0.05, **p < 0.01, ***p < 0.001

## Discussion

This is the most in-depth study of patients with NASH-related fibrosis for whom liver histology, collagen quantitation by morphometry, phosphoproteomic assessment of liver by RPMA, and serum proteomic assessment by LC–MS was performed. In this combined tissue and serum proteomics study of subjects with biopsy-proven NASH/NAFLD, we identified serum proteomic profiles as well as hepatic phosphoproteomic profiles that are associated with advanced fibrosis or increased hepatic collagen deposition in NASH.

Liver tissue from this cohort was used for RPMA to assess activated (phosphorylated) proteins in the hepatic tissue of subjects with NASH and advanced fibrosis. In this context, our data shows that the phosphorylated signaling proteins in the hepatic tissue that correlate with increased collagen deposition target the ASK1-MAPK pathway. Furthermore, activation of p38 MAPK T180/Y182 members of the MAPK family was noted. Notably, phosphorylation of p38 MAPK T180/Y182 participates in a signaling cascade that controls the cellular responses to inflammatory cytokines and stress [42]. It is also important to note that ASK1 S83 is an immunoregulatory protein that plays a pivotal role in apoptosis signaling, inflammation, and fibrosis in the setting of increased oxidative stress, associated with the pathogenesis of NASH. Therefore, inhibitors of the ASK1-MAPK pathway are thought to have potential as therapeutic targets in diseases where ASK1 is activated [43–47]. Our data support the concept that ASK1 activation is a key component for NASH-related fibrosis. In fact, a phase 2 clinical trial has been reported to target ASK1 inhibition as a potential treatment option for NASH. Early data from this study reported consistent improvement of hepatic fibrosis after a short course of treatment with an investigational ASK1 inhibitor in patients with NASH and fibrosis [47]. In concordance with these findings, our proteomics data from liver tissue, representative of organ-wide signaling, provides substantial evidence for the activation of the ASK pathway in the hepatic tissue of NASH subjects with advanced fibrosis, thus yielding further support for targeting of this pathway in the treatment of NAFLD patients with fibrosis.

In addition to the ASK pathway, our study also showed that IL-10 expression in the liver was associated with the presence of higher hepatic collagen deposition in NAFLD. IL-10 is a pleiotropic anti-inflammatory cytokine with important immunoregulatory functions [42]. In fact, IL-10 signaling pathway involves the phosphorylation and activation of a number of pathways that may be important in the pathogenesis of NASH and NASH-related fibrosis. These include the ASK1-MAPK pathway, the transducer activator transcription 3 and other survival pathways (insulin receptor substrate 2 via phosphoinositide-3 kinase class IA and its downstream effectors 3-phosphoinositide dependent protein kinase-1, ribosomal protein S6 kinase polypeptide 1, and v-Akt oncogene homolog) [48]. Despite these findings, the exact role of IL-10 and these pathways requires further investigation in larger matched cohorts.

The gene expression of NAFLD and alcoholic fatty liver disease is similar not only with respect to alcohol-metabolizing genes, but also with respect to numerous other genes, as shown by the global gene expression patterns observed. The demonstration of elevated expression of alcohol-metabolizing pathways in pediatric NASH livers [49] and alcohol-producing gut microbiomes in NASH livers [50] supports the hypothesis that endogenous gut-derived alcohol microbiomes could be a potential hit in the pathogenesis of NASH. Furthermore, the same group later [51] demonstrated that, in advanced stages of NAFLD, there is less expression of the alcohol-metabolizing genes compared to in mild NAFLD. Herein, we report a similar observation, where we found the expression of ALDH in livers to be negatively correlated with % collagen in the liver and negatively associated with fibrosis. We, and others [51], think that it is unexpected that the alcohol-metabolizing molecules are not associated with severe NAFLD. One justification for why these genes were not found to be expressed by fibrotic livers from NAFLD is because most of the alcohol-metabolizing molecules are related to hepatocytes, and there is higher hepatocyte damage in fibrotic livers of NAFLD. A further support for this explanation is that, in our study, NAFLD fibrotic livers showed higher expression of the apoptosis molecules than non-fibrotic livers from NAFLD. However, in our study, phosphoproteomics was performed on whole liver tissue, making it difficult to correlate the exact phosphoproteins specific to hepatocytes.

In addition to the liver tissue, we used sera from the same patients for high resolution MS, which has excellent analytical sensitivity, coupled to nanoparticle-based protein capture technology to concentrate low abundance but clinically relevant proteins [34, 35]. Using this approach, our data shows that a total of 19 circulating proteins are associated with either advanced fibrosis (stage ≥ 2) or higher percent hepatic collagen deposition. In fact, our multivariate analysis showed that serum α2M and coagulation factor V were independently associated with a higher % hepatic collagen deposition, confirming previous data [18], which suggests their potential role in hepatic collagen deposition in NASH. Furthermore, α2M strongly correlates with the downstream transcription factors Met Y1234/1235 and LIMK1 T508/LIMK2. These hepatic phosphoproteins have been independently associated with increased hepatic collagen, further connecting serum proteome to hepatic phosphoproteome and their potential roles in collagen deposition and hepatic fibrosis. Furthermore, these data may suggest that MET and LMK can be involved in the upstream pathogenic mechanism of fibrosis and collagen deposition and therefore the interplay of these molecules should be considered for further investigation.

In addition to the above findings, our data indicates that hypercoagulable factors may also play a role in the development and progression of hepatic fibrosis. The exact mechanism of this influence is not understood, but thrombin, through its activation by coagulation factor V, may be a key component [52, 53].

Liver plays a role in regulating glucose and lipid metabolism. Glucogenesis is suppressed with insulin resistance, which may lead to hyperglycemia resulting in hepatic steatosis [1, 54]. One of the major pathways implicated in hepatic function is the JAK/STAT pathway together with IL-6-STAT3 and IL-4-STAT6 pathways. Similarly, in our studies, we have shown that IL-6 and IL-10 were increased in patients with fibrosis, together with STAT4 and STAT6 (Additional file 1: Table S1). Previous studies have demonstrated that STAT3 absence in hepatocytes may result in insulin resistance and augmented expression of gluconeogenic genes, mediated by the dysregulation of IL-6 signaling [2, 3, 55, 56]. Taken together, this data support previous findings that STAT signaling is critical for liver metabolic functions. Dysregulation of STAT signaling pathways can result in disrupted hepatic glucose metabolism, leading to hepatic steatosis.

Finally, our systematic pathway analysis approach confirmed two key trends in the data. First, the majority of serum proteins discovered in this study seem to be best associated with the semi-quantitative assessment of advanced fibrosis by histopathology. In contrast, the phosphoproteins in the liver were more frequently associated with increased % hepatic collagen deposition as quantified by morphometry. Second, the data indicated that the proteins found to be independently associated with increased % hepatic collagen deposition are in pathways that are more intrinsically involved in the biology of fibrosis development. In contrast, proteins that are independent predictors of advanced fibrosis based on semi-quantitative assessment by histopathology are primarily involved in lipid storage and transport mechanisms. This suggests that proteins associated with increased % collagen deposition may be more sensitive markers of significant fibrosis in NASH.

**Fig. 1:**
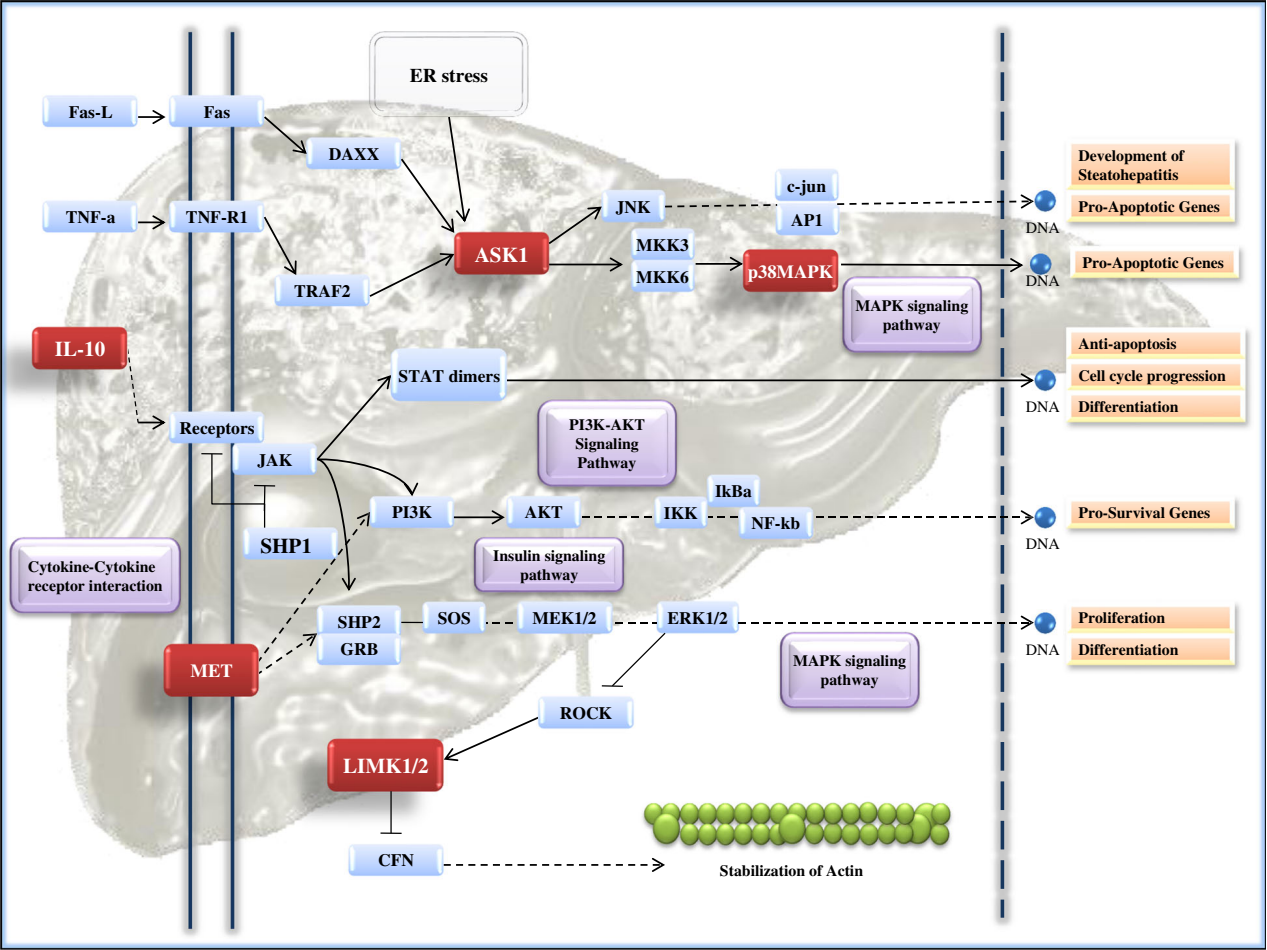
Composite pathway using the KEGG pathway analysis, a composite pathway was generated based on the canonical mapping of the phosphorylated proteins independently associated with increased % collagen (red) in the liver. Canonical pathway components are also indicated (blue) as well as direct and indirect transition pathways (purple)

The pathway distribution of IL-10 and ASK1 confirms that IL-10 is indeed upstream of the signaling activity of ASK1. It is notable that ASK1 independently mapped to both the NAFLD and apoptosis pathways (as delineated by KEGG) as this protein is known to be involved in hepatic steatosis and fibrosis [47]. In fact, when mapped to the apoptosis pathway, ASK1 is shown as upstream of pro-apoptotic genes and p38MAPK as upstream of pro-survival genes. In short, this pathway analysis confirms that ASK1 is indeed involved in the pathogenesis of hepatic fibrosis in NASH. Furthermore, the data indicates that, while IL-10 is an upstream effector, the signaling cascade does indeed pass through p38MAPK and seems to culminate with LIMK1/LIMK2 and its ability to block the stabilization of actin as seen in this composite pathway (Fig. 1).

There are some limitations to our study. First, it is an exploratory analysis of NASH and liver fibrosis processes and requires validation in a larger group of patients with NASH-related fibrosis. In fact, although in the context of multiple testing, the estimated false discovery rate was not very high; given the number of studied parameters and limited sample size, we could not rule out the risk of over-fitting for the presented models. Nevertheless, and to our knowledge, this is the first study utilizing a unique combination of tissue and serum samples coupled with proteomics analysis to identify serum proteome determined by MS- and RPMA-based protein pathway activation mapping of the liver tissue to uncover markers pertinent to fibrosis.

In summary, this study uses a very well described group of NASH subjects with advanced fibrosis with available serum and liver tissue. Our data indicates that ASK1-MAPK is the most important activated pathway in NASH subjects with advanced fibrosis. Additionally, our serum proteomic data confirms that α2M seems to have a predictive value for higher % hepatic collagen deposition in subjects with NASH. Our proteomics data (serum and hepatic) can provide guidance to investigators who are developing therapeutic targets for patients with a clinically relevant type of NAFLD, i.e., NASH with advanced fibrosis or steatofibrosis [57].

## Conclusion

NAFLD is the most common liver disease worldwide. NASH is a subtype of NAFLD that may progress to cirrhosis and other liver complications. Only fibrosis can independently predict liver-related mortality. Phosphorylated signaling pathways play a role in liver fibrosis, but none has been confirmed as a pathogenic mechanism and targets for NASH treatment are still under investigation. Using simultaneous profiling of sera proteome and phosphoproteome in liver tissue reveals that ASK1 S83, p38MAPK T180/Y182, the receptor tyrosine kinase, Met Y1234/1235, LIMK1T508/LIMK2 T505, and tissue remodeling-related inflammatory cytokines IL-10 were independently associated with higher collagen deposition in subjects with NAFLD. These pathogenic mechanisms have not been previously correlated to clinical diagnostic markers. However, we were able to show that α2M (a biomarker already implemented in the Fibro Sure test) is strongly correlated to ASK1 S38 and IL-10. Although the mechanism of action of these proteins is unclear, the data suggest a potential role for these proteins in the pathogenesis of fibrosis and potential therapeutic utility in patients with NASH. The phosphorylated signaling pathways that are independently correlated to fibrosis in NASH revealed by our study might well pave the way for the revelation of more therapeutic targets and contribute to understanding the pathogenic mechanism of fibrosis in NASH. Although in the context of multiple testing, the estimated false discovery rate was not very high given the number of studied parameters and limited sample size; however, we could not rule out the risk of over-fitting for the presented models. Future validation of potential biomarkers to better define the pathogenic mechanism and stratify progression of disease in NAFLD will have great clinical significance.

## Additional file

Additional file 1: **Figure S1.** Representative image of collagen quantification after staining with Sirius red. **Table S1.** Associations or correlations of phosphorylated hepatic proteins with fibrosis stage and higher % collagen deposition in livers of NAFLD patients. **Figure S2.** Images showing (a) advanced liver fibrosis signaling protein-protein network and (b) higher hepatic percentage collagen protein-protein network. **Figure S3.** Phosphoproteins and proteomes involved in biological processes and KEGG pathways. **Table S2.** Pathways associated with advanced fibrosis stage ≥ 2. **Table S3.** Pathways associated with higher % collagen deposition in the liver. **Figure S4.** Scatter plots of *A. alpha*-2 macroglobulin precursor vs. IL-10 (ρ = 0.28, *p* = 0.02) and ASK1 S83 (ρ = 0.25, *p* = 0.04). (DOCX 2995 kb)
