# Supplementary material for: An exploratory study examining how nano-liquid chromatography–mass spectrometry and phosphoproteomics can differentiate patients with advanced fibrosis and higher percentage collagen in non-alcoholic fatty liver disease
Source: BMC Med. 2018 Sep 12;16:170. doi: 10.1186/s12916-018-1136-1 (PMC6134795; doi:10.1186/s12916-018-1136-1)
Supplement: Supplementary file 1 — Figure S1. Representative image of collagen quantification after staining with Sirius red. Table S1. Associations or correlations of phosphorylated hepatic proteins with fibrosis stage and higher % collagen deposition in livers of NAFLD patients. Figure S2. Images showing (a) advanced liver fibrosis signaling protein-protein network and (b) higher hepatic percentage collagen protein-protein network. Figure S3. Phosphoproteins and proteomes involved in biological processes and KEGG pathways. Table S2. Pathways associated with advanced fibrosis stage ≥ 2. Table S3. Pathways associated with higher % collagen deposition in the liver. Figure S4. Scatter plots of A. alpha-2 macroglobulin precursor vs. IL-10 (ρ = 0.28, p = 0.02) and ASK1 S83 (ρ = 0.25, p = 0.04). (DOCX 2995 kb) [file 12916_2018_1136_MOESM1_ESM.docx]

**Figure S1** Representative image of collagen quantification after staining with Sirius red.

**
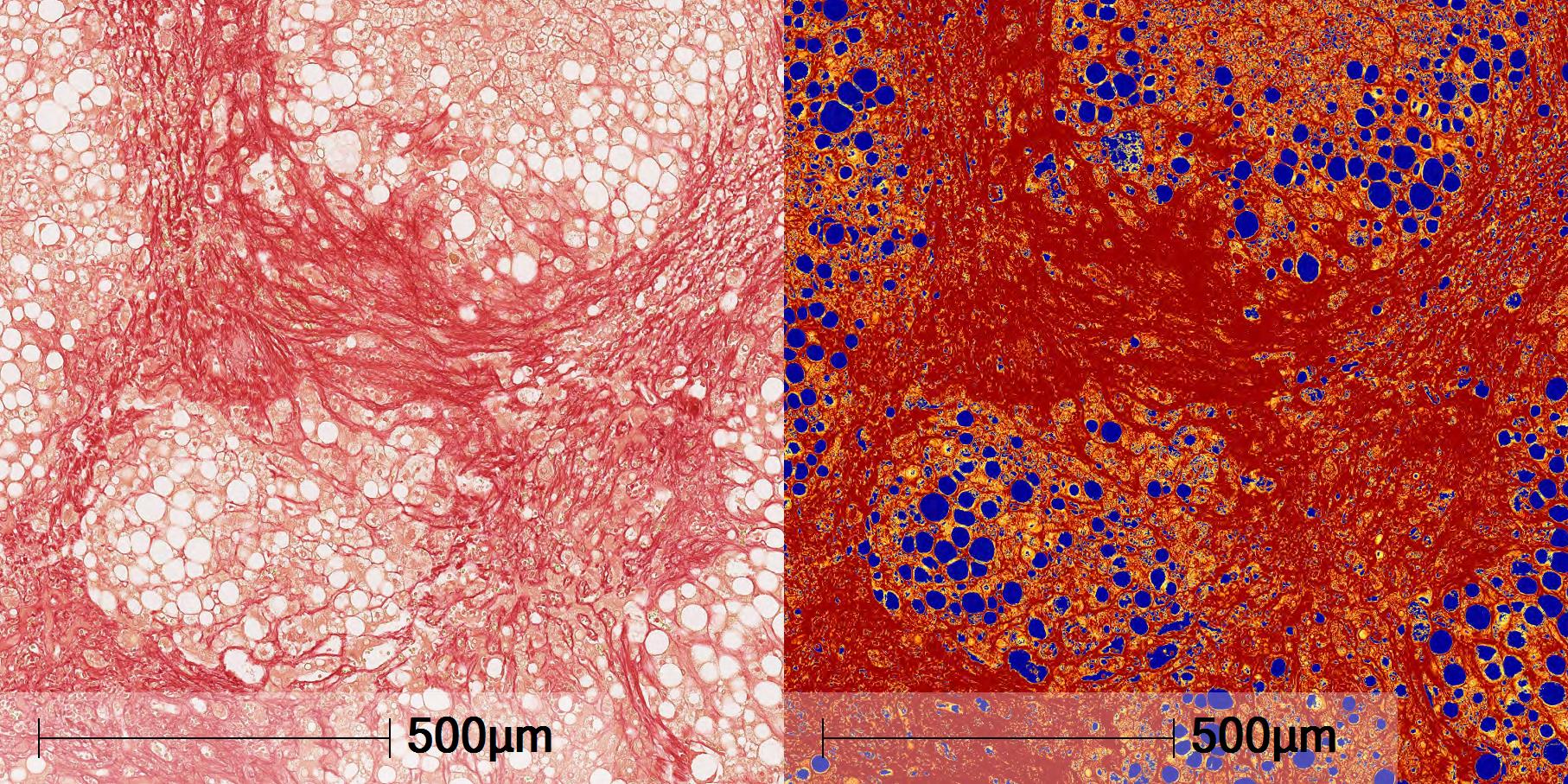
**

Note: The image to the left shows digitalized liver biopsy showing collagen fibrils stained with sirius red (magnification X8). The image to the right shows the quantification of this staining by computer-assisted morphometry expressed as percentage (%) collagen. The images are from a NASH patient diagnosed with fibrosis stage 3.

**Table S1** Associations or correlations of phosphorylated hepatic proteins with fibrosis stage and higher % collagen deposition in livers of NAFLD patients.

| **Endpoint** | **Protein Intensity Values**  **Mean)** | | | |  | | | | **% Collagen** | | |
| --- | --- | --- | --- | --- | --- | --- | --- | --- | --- | --- | --- |
|  | **Advanced fibrosis (Stage>2)** | **Mild**  **Fibrosis**  **(Stgae≤2)** | | | **p-value** | | | | **ρ** | | **p-value** |
| MITOGENESIS |  |  | | |  | | | |  | |  |
| RTKS and LIGANDS |  |  | | |  | | | |  | |  |
| c- Abl T735 | 13223.6069 | 10113.3845 | | | 0.0224 | | | | 0.1700 | | 0.1829 |
| c- Abl Y245 | 10551.0221 | 7758.1005 | | | 0.0292 | | | | 0.1394 | | 0.2760 |
| c- Kit Y703 | 12023.3253 | 8509.7594 | | | 0.0032 | | | | 0.2727 | | 0.0306 |
| c- Kit Y719 | 17434.7504 | 14551.3409 | | | 0.0050 | | | | 0.2069 | | 0.1037 |
| EGFR TOTAL | 15497.0934 | 11799.5914 | | | 0.0185 | | | | 0.2116 | | 0.0960 |
| EGFR Y1045 | 7752.7248 | 5367.3954 | | | 0.0059 | | | | 0.3061 | | 0.0147 |
| EGFR Y1068 | 16097.0728 | 11054.2309 | | | 0.0228 | | | | 0.1115 | | 0.3844 |
| EGFR Y1173 | 31458.7687 | 23455.3655 | | | 0.0003 | | | | 0.2261 | | 0.0748 |
| ErbB2 HER2 Y1248 | 13638.4722 | 10163.0936 | | | 0.0020 | | | | 0.2544 | | 0.0442 |
| ErbB3 HER3 | 6238.6107 | 4805.3851 | | | 0.0435 | | | | 0.1135 | | 0.3795 |
| ErbB4 HER4 111B2 | 8310.5635 | 7132.6174 | | | 0.0470 | | | | 0.2063 | | 0.1076 |
| Estrogen Rec alpha S118 | 13588.9026 | 11165.2081 | | | 0.0169 | | | | 0.2307 | | 0.0712 |
| IGF I Receptor beta Y1131/Insulin Receptor beta Y1146 | 18604.5885 | 16123.1557 | | | 0.0044 | | | | 0.1657 | | 0.1981 |
| FGFR Y653 654 | 13172.8317 | 10860.7056 | | | 0.0500 | | | | 0.2292 | | 0.0732 |
| Heregulin | 12801.0701 | 10285.1745 | | | 0.0123 | | | | 0.2393 | | 0.0611 |
| Met Y1234/1235 | 8394.0971 | 5442.5366 | | | 0.0013 | | | | 0.4082 | | 0.0010 |
| PDGFRb Y716 | 13804.4777 | 11787.9870 | | | 0.0136 | | | | 0.1316 | | 0.3081 |
| PDGFRb Y751 | 5643.7596 | 3794.5438 | | | 0.0141 | | | | 0.1707 | | 0.1848 |
| Ret Y905 | 4840.5597 | 2796.3384 | | | 0.0003 | | | | 0.2564 | | 0.0442 |
| Ron Y1353 | 11455.9458 | 8217.8116 | | | 0.0096 | | | | 0.2564 | | 0.0442 |
| VEGFR2 Y1175 | 11185.7993 | 7467.1069 | | | 0.0096 | | | | 0.2239 | | 0.0802 |
| DOWNSTREAM SUBSTRATES | | | | | | | | | | | |
| ATF 2 T71 | 9510.6540 | | | 6013.5346 | | | 0.0078 | | | 0.2025 | 0.1144 |
| a Raf S299 | 2036.7795 | | | 2761.4601 | | | 0.0445 | | | 0.1209 | 0.3492 |
| CrkII Y221 | 11296.7507 | | | 7283.2336 | | | 0.0009 | | | 0.2844 | 0.0251 |
| c- Myc | 16583.2182 | | | 13624.0484 | | | 0.0267 | | | 0.2705 | 0.0335 |
| ERK 1/2 TOTAL | 27683.0657 | | | 22584.3185 | | | 0.0037 | | | 0.3574 | 0.0044 |
| FRS2 alpha Y436 | 6745.9092 | | | 4569.8235 | | | 0.0389 | | | 0.3382 | 0.0072 |
| IRS 1 S612 | 6603.2819 | | | 4223.5915 | | | 0.0353 | | | 0.2756 | 0.0301 |
| MARCKS S152/156 | 8269.1435 | | | 6103.1889 | | | 0.0294 | | | 0.2379 | 0.0626 |
| PKA C T197 | 13849.3962 | | | 11729.8932 | | | 0.0428 | | | 0.2968 | 0.0192 |
| PKC delta T505 | 12828.7635 | | | 9636.6193 | | | 0.0143 | | | 0.2423 | 0.0577 |
| PKC theta T538 | 4835.6505 | | | 2880.2304 | | | 0.0028 | | | 0.1515 | 0.2399 |
| Shc Y317 | 11255.8745 | | | 7640.6374 | | | 0.0044 | | | 0.1875 | 0.1445 |
| Smad2 S465 467 | 12774.9902 | | | 10429.0775 | | | 0.0111 | | | 0.0737 | 0.5694 |
| Src Family Y416 | 3488.0194 | | | 1874.4209 | | | 0.0021 | | | 0.2631 | 0.0389 |
| Src Y527 | 15938.5775 | | | 14210.1954 | | | 0.0421 | | | 0.1837 | 0.1531 |
| S6 Ribosomal protein S235/236 | 3692.8200 | | | 2006.6400 | | | 0.0900 | | | 0.2770 | 0.0293 |
| SURVIVAL | | | | | | | | | | | |
| AKT T308 | 19966.2896 | | | 11089.2265 | | | 0.0021 | | | 0.3763 | 0.0026 |
| AKT TOTAL | 15739.0600 | | | 10127.1820 | | | 0.0057 | | | 0.3282 | 0.0092 |
| ASK1 S83 | 2072.4277 | | | 1209.4175 | | | 0.0253 | | | 0.2563 | 0.0443 |
| eNOS S113 | 6266.0753 | | | 4975.1319 | | | 0.0146 | | | 0.0996 | 0.4414 |
| Ephrin A3 Y779/A4 Y779/A5 Y833 | 10051.2853 | | | 9008.6468 | | | 0.0133 | | | 0.3179 | 0.0118 |
| PDK1 S241 | 13526.9204 | | | 10692.6456 | | | 0.0016 | | | 0.2990 | 0.0182 |
| PI3K p85 Y458 p55 Y199 | 5930.4957 | | | 4023.6630 | | | 0.0017 | | | 0.4243 | 0.0006 |
| PTEN | 26544.6698 | | | 22904.4300 | | | 0.0232 | | | 0.2628 | 0.0390 |
| PTEN S380 | 12498.2321 | | | 7720.4331 | | | 0.0006 | | | 0.2677 | 0.0354 |
| Pyk2 Y402 | 11619.3583 | | | 7861.6006 | | | 0.0039 | | | 0.2077 | 0.1053 |
| RSK3 T356 S360 | 11490.4020 | | | 7892.6054 | | | 0.0202 | | | 0.2358 | 0.0650 |
| SGK1 S78 | 18462.5136 | | | 16100.6469 | | | 0.0266 | | | 0.1818 | 0.1573 |
| ATP Citrate Lyase S454 | 7731.9600 | | | 6009.3300 | | | 0.1300 | | | 0.3486 | 0.0055 |
| INFLAMMATION/IMMUNE FUNCTION | | | | | | | | | | | |
| SEK1/MKK4 S80 | 11984.1691 | | 9774.8174 | | | 0.0492 | | 0.1869 | | | 0.1458 |
| Zap 70 Y319/Syk Y352 | 8322.9034 | | 4839.8932 | | | 0.0003 | | 0.3189 | | | 0.0115 |
| cPLA2 S505 | 11697.1946 | | 8108.4437 | | | 0.0034 | | 0.2861 | | | 0.0242 |
| PLCgamma1 Y783 | 11995.7606 | | 8488.3315 | | | 0.0067 | | 0.2824 | | | 0.0262 |
| PRK1 T774/PRK2 T816 | 12859.8790 | | 9262.0615 | | | 0.0129 | | 0.2192 | | | 0.0869 |
| Stat1 Y701 | 19059.3621 | | 17555.0009 | | | 0.0395 | | 0.1264 | | | 0.3275 |
| Stat2 Y690 | 7940.5823 | | 5601.8650 | | | 0.0081 | | 0.1355 | | | 0.2937 |
| Stat4 Y693 | 8407.7191 | | 5859.6088 | | | 0.0024 | | 0.3010 | | | 0.0174 |
| Stat5 Y694 | 18671.9609 | | 13224.2955 | | | 0.0258 | | 0.2231 | | | 0.0813 |
| Stat6 Y641 | 7840.7122 | | 4793.4883 | | | 0.0004 | | 0.3226 | | | 0.0106 |
| Tyk2 Y1054/1055 | 8760.8030 | | 5060.9800 | | | 0.0001 | | 0.2955 | | | 0.0197 |
| VASP S157 | 11432.3742 | | 8606.3219 | | | 0.0168 | | 0.1745 | | | 0.1749 |
| Vav3 Y173 | 10971.9202 | | 7846.2598 | | | 0.0199 | | 0.1177 | | | 0.3622 |
| p38 MAPK T180/Y182 | 8093.9200 | | 6807.1800 | | | 0.6300 | | 0.3901 | | | 0.0017 |
| AUTOPHAGY |  | |  | | |  | |  | | |  |
| Beclin 1 | 11546.8048 | | 9282.5367 | | | 0.0151 | | 0.2345 | | | 0.0666 |
| LC3B | 8577.9109 | | 6767.6769 | | | 0.0129 | | 0.2302 | | | 0.0719 |
| ATG12 | 2733.9300 | | 2153.2400 | | | 0.1500 | | 0.3074 | | | 0.0151 |
| APOPTOSIS |  | |  | | |  | |  | | |  |
| BAD S136 | 10426.6499 | | 7976.1778 | | | 0.0331 | | 0.1994 | | | 0.1203 |
| Bak | 20974.0624 | | 18147.0249 | | | 0.0037 | | 0.3262 | | | 0.0097 |
| Bax | 11347.7590 | | 8655.1653 | | | 0.0232 | | 0.1973 | | | 0.1243 |
| Bcl 2 S70 | 16596.1015 | | 14425.5395 | | | 0.0213 | | 0.1879 | | | 0.1436 |
| Survivin | 11593.7421 | | 8882.5924 | | | 0.0005 | | 0.3180 | | | 0.0118 |
| XIAP | 10965.4166 | | 7598.1326 | | | 0.0302 | | 0.1794 | | | 0.1629 |
| HSP27 S82 | 15633.9200 | | 13912.5100 | | | 0.2300 | | 0.2983 | | | 0.0185 |
| MOTILITY AND CELL ADHESION |  | |  | | |  | |  | | |  |
| Cofilin S3 | 11887.6509 | | 2171.5929 | | | 0.0000 | | 0.3369 | | | 0.0074 |
| FAK Y576/577 | 7300.4566 | | 5303.6335 | | | 0.0175 | | 0.3382 | | | 0.0072 |
| GRB2 | 14636.1000 | | 13053.9600 | | | 0.0900 | | 0.2978 | | | 0.0187 |
| CELL CYCLE CONTROL |  | |  | | |  | |  | | |  |
| PLK1 T210 | 21513.8844 | | 19420.0859 | | | 0.0415 | | 0.2119 | | | 0.0983 |
| YAP S127 | 16403.0826 | | 12597.9342 | | | 0.0096 | | 0.2460 | | | 0.0540 |
| MDM2 S166 | 6382.0900 | | 5264.1200 | | | 0.8800 | | 0.2614 | | | 0.0402 |
| STROMA PROTEIN |  | |  | | |  | |  | | |  |
| TWIST | 3880.3784 | | 2216.5695 | | | 0.0048 | | 0.1326 | | | 0.3042 |
| Vimentin | 11643.9254 | | 7938.8923 | | | 0.0047 | | 0.2223 | | | 0.0825 |
| OTHERS |  | |  | | |  | |  | | |  |
| ALDH | 21453.9429 | | 26006.4687 | | | 0.0063 | | -0.3009 | | | 0.0175 |
| IL-6 | 9315.7700 | | 8546.4400 | | | 0.2300 | | 0.2909 | | | 0.0218 |
| IL-10 | 1731.1300 | | 1311.5200 | | | 0.1400 | | 0.5539 | | | 0.0000 |

**Figure S2 Images showing (a) advanced liver fibrosis signaling protein-protein network (b) higher hepatic percentage collagen protein-protein network.** Representation of the signaling network interaction of phosphorylated proteins measured by RPPA that reached a correlation coefficient greater than 0.9. Interactions detected in the liver tissues of patients with advanced fibrosis and collagen >5.36% are shown in Panel A and B respectively. Interconnection between proteins involved in the neoangiogenesis, cell motility, and immune response were detected across the two groups.

**
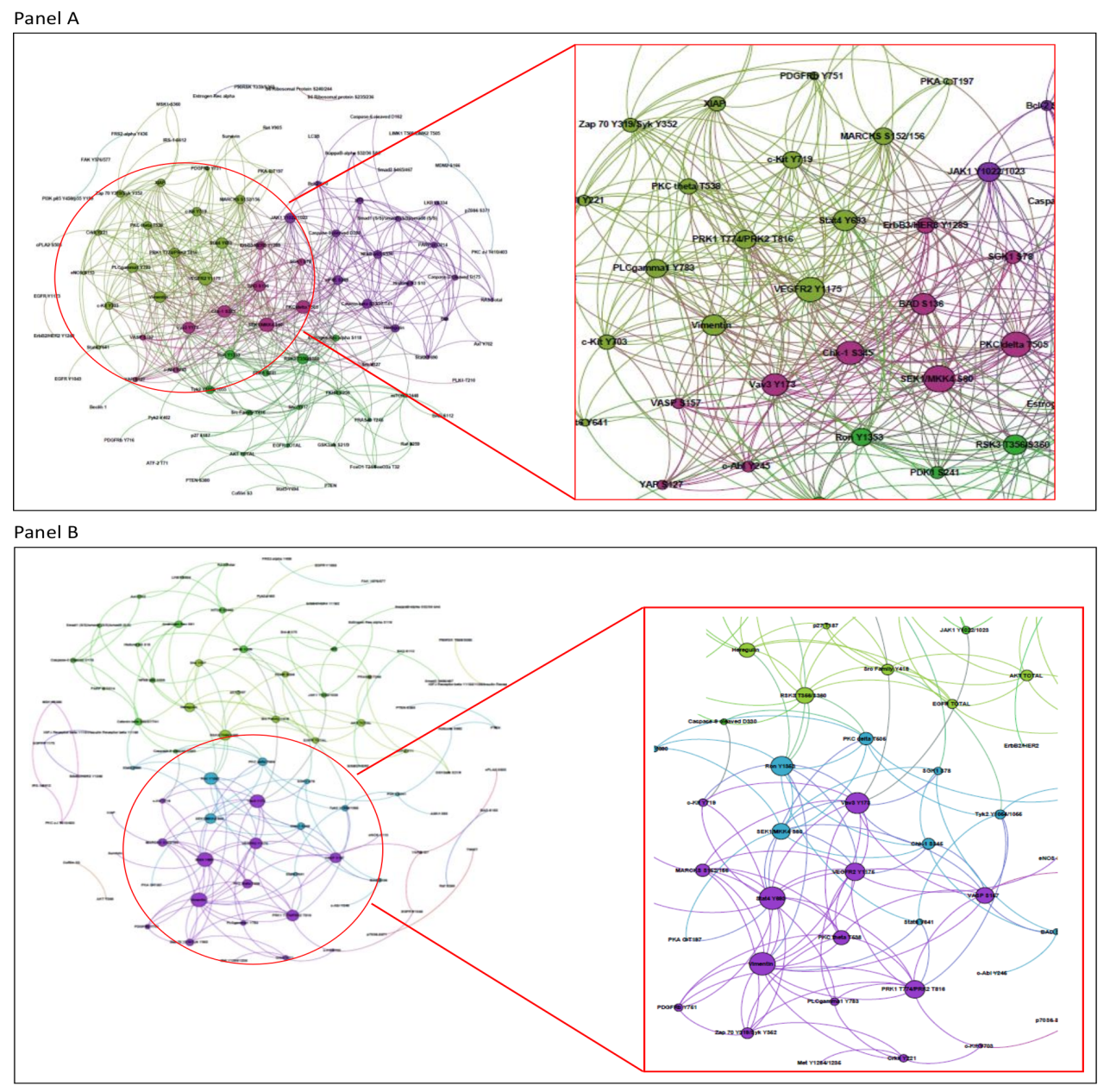
**

**Figure S3** P**hosphoproteins and proteomes involved in biological processes and KEGG pathways.** 2A. Molecules found to be independent predictors of Advanced Fibrosis (Fibrosis ≥ 2) and their respective pathways. 2B. Molecules found to be independent predictors of % Collagen and their respective pathways.


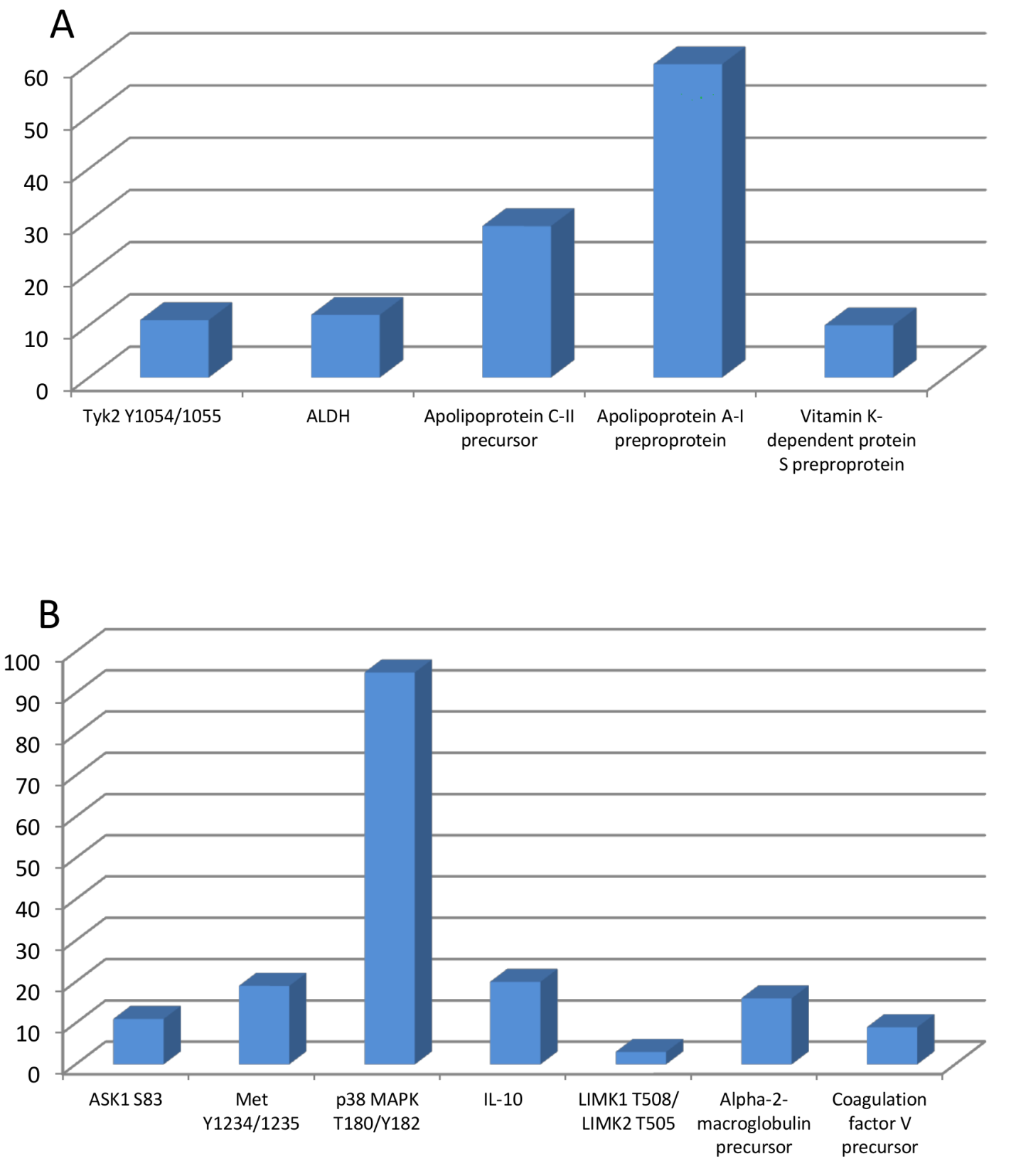


**Table S2** Pathways associated with Advanced Fibrosis Stage>2

| **Protein** | **ID** | **KEGG Pathways and Biological Processes** | **Enzyme and pathway databases** | **Number of Pathways** |
| --- | --- | --- | --- | --- |
| **Tyk2 Y1054/1055** | 7297 | 1. hsa05145 Toxoplasmosis - Homo sapiens (human) (1) 2. hsa04621 NOD-like receptor signaling pathway - Homo sapiens (human) (1) 3. hsa04658 Th1 and Th2 cell differentiation - Homo sapiens (human) (1) 4. hsa05160 Hepatitis C - Homo sapiens (human) (1) 5. hsa04380 Osteoclast differentiation - Homo sapiens (human) (1) 6. hsa05169 Epstein-Barr virus infection - Homo sapiens (human) (1) 7. hsa04659 Th17 cell differentiation - Homo sapiens (human) (1) 8. hsa05162 Measles - Homo sapiens (human) (1) 9. hsa04630 Jak-STAT signaling pathway - Homo sapiens (human) (1) 10. hsa05168 Herpes simplex infection - Homo sapiens (human) (1) 11. hsa05164 Influenza A - Homo sapiens (human) (1) |  | 11 |
| **ALDH** | 217 | 1. hsa00310 Lysine degradation - Homo sapiens (human) (1) 2. hsa01100 Metabolic pathways - Homo sapiens (human) (1) 3. hsa00561 Glycerolipid metabolism - Homo sapiens (human) (1) 4. hsa00620 Pyruvate metabolism - Homo sapiens (human) (1) 5. hsa00340 Histidine metabolism - Homo sapiens (human) (1) 6. hsa00280 Valine, leucine and isoleucine degradation - Homo sapiens (human) (1) 7. hsa00410 beta-Alanine metabolism - Homo sapiens (human) (1) 8. hsa00071 Fatty acid degradation - Homo sapiens (human) (1) 9. hsa00330 Arginine and proline metabolism - Homo sapiens (human) (1) 10. hsa00010 Glycolysis / Gluconeogenesis - Homo sapiens (human) (1) 11. hsa00053 Ascorbate and aldarate metabolism - Homo sapiens (human) (1) 12. hsa00380 Tryptophan metabolism - Homo sapiens (human) (1) |  | 12 |
| **Apolipoprotein C-II precursor** | P02655 | 1. lipase inhibitor activity Source: BHF-UCL 2. lipid binding Source: BHF-UCL 3. lipoprotein lipase activator activity Source: BHF-UCL 4. phospholipase activator activity Source: BHF-UCL 5. phospholipase binding Source: BHF-UCL 6. protein homodimerization activity Source: BHF-UCL 7. cholesterol efflux Source: BHF-UCL 8. cholesterol homeostasis Source: BHF-UCL 9. chylomicron remnant clearance Source: BHF-UCL 10. chylomicron remodeling Source: BHF-UCL 11. high-density lipoprotein particle clearance Source: BHF-UCL 12. lipid catabolic process Source: UniProtKB-KW 13. lipoprotein metabolic process Source: Reactome 14. negative regulation of cholesterol transport Source: BHF-UCL 15. negative regulation of lipid metabolic process Source: BHF-UCL 16. negative regulation of receptor-mediated endocytosis Source: BHF-UCL 17. negative regulation of very-low-density lipoprotein particle clearance Source: BHF-UCL 18. phospholipid efflux Source: BHF-UCL 19. positive regulation of fatty acid biosynthetic process Source: BHF-UCL 20. positive regulation of lipoprotein lipase activity Source: BHF-UCL 21. positive regulation of phospholipase activity Source: BHF-UCL 22. positive regulation of phospholipid catabolic process Source: BHF-UCL 23. positive regulation of triglyceride catabolic process Source: BHF-UCL 24. positive regulation of very-low-density lipoprotein particle remodeling Source: BHF-UCL 25. retinoid metabolic process Source: Reactome 26. reverse cholesterol transport Source: BHF-UCL 27. triglyceride homeostasis Source: BHF-UCL 28. triglyceride-rich lipoprotein particle remodeling Source: BHF-UCL 29. very-low-density lipoprotein particle remodeling Source: BHF-UCL | R-HSA-174800. Chylomicron-mediated lipid transport.  R-HSA-194223. HDL-mediated lipid transport.  R-HSA-975634. Retinoid metabolism and transport. | 29 |
| **Apolipoprotein A-I preproprotein** | P02647 | 1. adrenal gland development Source: Ensembl 2. animal organ regeneration Source: Ensembl 3. blood vessel endothelial cell migration Source: Ensembl 4. cellular protein metabolic process Source: Reactome 5. cholesterol biosynthetic process Source: GO_Central 6. cholesterol efflux Source: BHF-UCL 7. cholesterol homeostasis Source: BHF-UCL 8. cholesterol import Source: BHF-UCL 9. cholesterol metabolic process Source: BHF-UCL 10. cholesterol transport Source: MGI 11. endothelial cell proliferation Source: Ensembl 12. glucocorticoid metabolic process Source: Ensembl 13. G-protein coupled receptor signaling pathway Source: BHF-UCL 14. high-density lipoprotein particle assembly Source: BHF-UCL 15. high-density lipoprotein particle clearance Source: BHF-UCL 16. high-density lipoprotein particle remodeling Source: BHF-UCL 17. integrin-mediated signaling pathway Source: UniProtKB 18. lipid storage Source: Ensembl 19. lipoprotein biosynthetic process Source: Reactome 20. lipoprotein metabolic process Source: GO_Central 21. negative chemotaxis Source: UniProtKB 22. negative regulation of cell adhesion molecule production Source: BHF-UCL 23. negative regulation of cytokine secretion involved in immune response Source: BHF-UCL 24. negative regulation of heterotypic cell-cell adhesion Source: BHF-UCL 25. negative regulation of inflammatory response Source: BHF-UCL 26. negative regulation of interleukin-1 beta secretion Source: BHF-UCL 27. negative regulation of lipase activity Source: Ensembl 28. negative regulation of response to cytokine stimulus Source: BHF-UCL 29. negative regulation of tumor necrosis factor-mediated signaling pathway Source: BHF-UCL 30. negative regulation of very-low-density lipoprotein particle remodeling Source: BHF-UCL 31. neuron projection regeneration Source: GO_Central 32. peptidyl-methionine modification Source: UniProtKB 33. peripheral nervous system axon regeneration Source: Ensembl 34. phosphatidylcholine biosynthetic process Source: BHF-UCL 35. phospholipid efflux Source: BHF-UCL 36. phospholipid homeostasis Source: BHF-UCL 37. platelet degranulation Source: Reactome 38. positive regulation of cholesterol esterification Source: BHF-UCL 39. positive regulation of fatty acid biosynthetic process Source: GO_Central 40. positive regulation of hydrolase activity Source: BHF-UCL 41. positive regulation of lipoprotein lipase activity Source: GO_Central 42. positive regulation of Rho protein signal transduction Source: UniProtKB 43. positive regulation of stress fiber assembly Source: UniProtKB 44. positive regulation of substrate adhesion-dependent cell spreading Source: UniProtKB 45. positive regulation of triglyceride catabolic process Source: GO_Central 46. protein oxidation Source: UniProtKB 47. protein stabilization Source: BHF-UCL 48. receptor-mediated endocytosis Source: Reactome 49. regulation of Cdc42 protein signal transduction Source: BHF-UCL 50. regulation of intestinal cholesterol absorption Source: GO_Central 51. regulation of protein phosphorylation Source: Ensembl 52. response to drug Source: Ensembl 53. response to estrogen Source: Ensembl 54. response to nutrient Source: Ensembl 55. retinoid metabolic process Source: Reactome 56. reverse cholesterol transport Source: BHF-UCL 57. transmembrane transport Source: Reactome 58. triglyceride catabolic process Source: GO_Central 59. triglyceride homeostasis Source: BHF-UCL 60. vitamin transport Source: AgBase | R-HSA-114608. Platelet degranulation.  R-HSA-1369062. ABC transporters in lipid homeostasis.  R-HSA-174800. Chylomicron-mediated lipid transport.  R-HSA-194223. HDL-mediated lipid transport.  R-HSA-1989781. PPARA activates gene expression.  R-HSA-2168880. Scavenging of heme from plasma.  R-HSA-3000471. Scavenging by Class B Receptors.  R-HSA-3000480. Scavenging by Class A Receptors.  R-HSA-975634. Retinoid metabolism and transport.  R-HSA-977225. Amyloid fiber formation. | 60 |
| **vitamin K-dependent protein S preproprotein** | P07225 | 1. calcium ion binding Source: InterPro 2. endopeptidase inhibitor activity Source: ProtInc 3. blood coagulation Source: Reactome 4. ER to Golgi vesicle-mediated transport Source: Reactome 5. fibrinolysis Source: UniProtKB-KW 6. leukocyte migration Source: Reactome 7. peptidyl-glutamic acid carboxylation Source: Reactome 8. platelet degranulation Source: Reactome 9. regulation of complement activation Source: Reactome 10. signal peptide processing Source: Reactome | R-HSA-114608. Platelet degranulation.  R-HSA-140875. Common Pathway of Fibrin Clot Formation.  R-HSA-159740. Gamma-carboxylation of protein precursors.  R-HSA-159763. Transport of gamma-carboxylated protein precursors from the endoplasmic reticulum to the Golgi apparatus.  R-HSA-159782. Removal of aminoterminal propeptides from gamma-carboxylated proteins.  R-HSA-202733. Cell surface interactions at the vascular wall.  R-HSA-977606. Regulation of Complement cascade. | 10 |
| **Total** |  |  |  | 122 |

**Supplementary Table 3.** Pathways Associated with Higher % Collagen Deposition in the Liver

| **Protein** | **ID** | **KEGG Pathways and Biological Processes** | **Enzyme and pathway databases** | **Number of Pathways** |
| --- | --- | --- | --- | --- |
| **ASK1 S83** | 4217 | 1. hsa04530 Tight junction - Homo sapiens (human) (1) 2. hsa04210 Apoptosis - Homo sapiens (human) (1) 3. hsa05014 Amyotrophic lateral sclerosis (ALS) - Homo sapiens (human) (1) 4. hsa04010 MAPK signaling pathway - Homo sapiens (human) (1) 5. hsa04141 Protein processing in endoplasmic reticulum - Homo sapiens (human) (1) 6. hsa05418 Fluid shear stress and atherosclerosis - Homo sapiens (human) (1) 7. hsa04932 Non-alcoholic fatty liver disease (NAFLD) - Homo sapiens (human) (1) 8. hsa04668 TNF signaling pathway - Homo sapiens (human) (1) 9. hsa01524 Platinum drug resistance - Homo sapiens (human) (1) 10. hsa04722 Neurotrophin signaling pathway - Homo sapiens (human) (1) 11. hsa04071 Sphingolipid signaling pathway - Homo sapiens (human) (1) |  |  |
| **Met Y1234/1235** | 4233 | 1. hsa05120 Epithelial cell signaling in Helicobacter pylori infection - Homo sapiens (human) (1) 2. hsa05100 Bacterial invasion of epithelial cells - Homo sapiens (human) (1) 3. hsa05144 Malaria - Homo sapiens (human) (1) 4. hsa04151 PI3K-Akt signaling pathway - Homo sapiens (human) (1) 5. hsa05206 MicroRNAs in cancer - Homo sapiens (human) (1) 6. hsa04015 Rap1 signaling pathway - Homo sapiens (human) (1) 7. hsa04060 Cytokine-cytokine receptor interaction - Homo sapiens (human) (1) 8. hsa05211 Renal cell carcinoma - Homo sapiens (human) (1) 9. hsa05230 Central carbon metabolism in cancer - Homo sapiens (human) (1) 10. hsa01521 EGFR tyrosine kinase inhibitor resistance - Homo sapiens (human) (1) 11. hsa04510 Focal adhesion - Homo sapiens (human) (1) 12. hsa04144 Endocytosis - Homo sapiens (human) (1) 13. hsa05200 Pathways in cancer - Homo sapiens (human) (1) 14. hsa04014 Ras signaling pathway - Homo sapiens (human) (1) 15. hsa04520 Adherens junction - Homo sapiens (human) (1) 16. hsa05205 Proteoglycans in cancer - Homo sapiens (human) (1) 17. hsa05202 Transcriptional misregulation in cancer - Homo sapiens (human) (1) 18. hsa05218 Melanoma - Homo sapiens (human) (1) 19. hsa04360 Axon guidance - Homo sapiens (human) (1) |  |  |
| **p38 MAPK T180/Y182** | 5594 | 1. hsa05160 Hepatitis C - Homo sapiens (human) (1) 2. hsa04930 Type II diabetes mellitus - Homo sapiens (human) (1) 3. hsa04810 Regulation of actin cytoskeleton - Homo sapiens (human) (1) 4. hsa01522 Endocrine resistance - Homo sapiens (human) (1) 5. hsa05205 Proteoglycans in cancer - Homo sapiens (human) (1) 6. hsa05200 Pathways in cancer - Homo sapiens (human) (1) 7. hsa05132 Salmonella infection - Homo sapiens (human) (1) 8. hsa05145 Toxoplasmosis - Homo sapiens (human) (1) 9. hsa04726 Serotonergic synapse - Homo sapiens (human) (1) 10. hsa04210 Apoptosis - Homo sapiens (human) (1) 11. hsa04261 Adrenergic signaling in cardiomyocytes - Homo sapiens (human) (1) 12. hsa04611 Platelet activation - Homo sapiens (human) (1) 13. hsa04520 Adherens junction - Homo sapiens (human) (1) 14. hsa04666 Fc gamma R-mediated phagocytosis - Homo sapiens (human) (1) 15. hsa04914 Progesterone-mediated oocyte maturation - Homo sapiens (human) (1) 16. hsa05142 Chagas disease (American trypanosomiasis) - Homo sapiens (human) (1) 17. hsa04151 PI3K-Akt signaling pathway - Homo sapiens (human) (1) 18. hsa04713 Circadian entrainment - Homo sapiens (human) (1) 19. hsa05223 Non-small cell lung cancer - Homo sapiens (human) (1) 20. hsa04724 Glutamatergic synapse - Homo sapiens (human) (1) 21. hsa05164 Influenza A - Homo sapiens (human) (1) 22. hsa04371 Apelin signaling pathway - Homo sapiens (human) (1) 23. hsa04730 Long-term depression - Homo sapiens (human) (1) 24. hsa05216 Thyroid cancer - Homo sapiens (human) (1) 25. hsa04664 Fc epsilon RI signaling pathway - Homo sapiens (human) (1) 26. hsa04350 TGF-beta signaling pathway - Homo sapiens (human) (1) 27. hsa05213 Endometrial cancer - Homo sapiens (human) (1) 28. hsa04960 Aldosterone-regulated sodium reabsorption - Homo sapiens (human) (1) 29. hsa04072 Phospholipase D signaling pathway - Homo sapiens (human) (1) 30. hsa05218 Melanoma - Homo sapiens (human) (1) 31. hsa04510 Focal adhesion - Homo sapiens (human) (1) 32. hsa04071 Sphingolipid signaling pathway - Homo sapiens (human) (1) 33. hsa04022 cGMP-PKG signaling pathway - Homo sapiens (human) (1) 34. hsa04725 Cholinergic synapse - Homo sapiens (human) (1) 35. hsa04014 Ras signaling pathway - Homo sapiens (human) (1) 36. hsa04919 Thyroid hormone signaling pathway - Homo sapiens (human) (1) 37. hsa04550 Signaling pathways regulating pluripotency of stem cells - Homo sapiens (human) (1) 38. hsa05214 Glioma - Homo sapiens (human) (1) 39. hsa04662 B cell receptor signaling pathway - Homo sapiens (human) (1) 40. hsa05034 Alcoholism - Homo sapiens (human) (1) 41. hsa05221 Acute myeloid leukemia - Homo sapiens (human) (1) 42. hsa04621 NOD-like receptor signaling pathway - Homo sapiens (human) (1) 43. hsa04723 Retrograde endocannabinoid signaling - Homo sapiens (human) (1) 44. hsa05219 Bladder cancer - Homo sapiens (human) (1) 45. hsa05211 Renal cell carcinoma - Homo sapiens (human) (1) 46. hsa04150 mTOR signaling pathway - Homo sapiens (human) (1) 47. hsa05206 MicroRNAs in cancer - Homo sapiens (human) (1) 48. hsa04066 HIF-1 signaling pathway - Homo sapiens (human) (1) 49. hsa04360 Axon guidance - Homo sapiens (human) (1) 50. hsa04270 Vascular smooth muscle contraction - Homo sapiens (human) (1) 51. hsa05215 Prostate cancer - Homo sapiens (human) (1) 52. hsa04912 GnRH signaling pathway - Homo sapiens (human) (1) 53. hsa01521 EGFR tyrosine kinase inhibitor resistance - Homo sapiens (human) (1) 54. hsa05131 Shigellosis - Homo sapiens (human) (1) 55. hsa04320 Dorso-ventral axis formation - Homo sapiens (human) (1) 56. hsa04933 AGE-RAGE signaling pathway in diabetic complications - Homo sapiens (human) (1) 57. hsa04668 TNF signaling pathway - Homo sapiens (human) (1) 58. hsa04915 Estrogen signaling pathway - Homo sapiens (human) (1) 59. hsa04380 Osteoclast differentiation - Homo sapiens (human) (1) 60. hsa05010 Alzheimer's disease - Homo sapiens (human) (1) 61. hsa05020 Prion diseases - Homo sapiens (human) (1) 62. hsa04720 Long-term potentiation - Homo sapiens (human) (1) 63. hsa05224 Breast cancer - Homo sapiens (human) (1) 64. hsa04659 Th17 cell differentiation - Homo sapiens (human) (1) 65. hsa04910 Insulin signaling pathway - Homo sapiens (human) (1) 66. hsa04917 Prolactin signaling pathway - Homo sapiens (human) (1) 67. hsa05152 Tuberculosis - Homo sapiens (human) (1) 68. hsa04114 Oocyte meiosis - Homo sapiens (human) (1) 69. hsa04657 IL-17 signaling pathway - Homo sapiens (human) (1) 70. hsa04620 Toll-like receptor signaling pathway - Homo sapiens (human) (1) 71. hsa04921 Oxytocin signaling pathway - Homo sapiens (human) (1) 72. hsa05140 Leishmaniasis - Homo sapiens (human) (1) 73. hsa04722 Neurotrophin signaling pathway - Homo sapiens (human) (1) 74. hsa04068 FoxO signaling pathway - Homo sapiens (human) (1) 75. hsa04015 Rap1 signaling pathway - Homo sapiens (human) (1) 76. hsa05230 Central carbon metabolism in cancer - Homo sapiens (human) (1) 77. hsa04010 MAPK signaling pathway - Homo sapiens (human) (1) 78. hsa04916 Melanogenesis - Homo sapiens (human) (1) 79. hsa04140 Autophagy - animal - Homo sapiens (human) (1) 80. hsa05231 Choline metabolism in cancer - Homo sapiens (human) (1) 81. hsa04658 Th1 and Th2 cell differentiation - Homo sapiens (human) (1) 82. hsa05161 Hepatitis B - Homo sapiens (human) (1) 83. hsa04370 VEGF signaling pathway - Homo sapiens (human) (1) 84. hsa05212 Pancreatic cancer - Homo sapiens (human) (1) 85. hsa04650 Natural killer cell mediated cytotoxicity - Homo sapiens (human) (1) 86. hsa05203 Viral carcinogenesis - Homo sapiens (human) (1) 87. hsa04024 cAMP signaling pathway - Homo sapiens (human) (1) 88. hsa04660 T cell receptor signaling pathway - Homo sapiens (human) (1) 89. hsa05133 Pertussis - Homo sapiens (human) (1) 90. hsa01524 Platinum drug resistance - Homo sapiens (human) (1) 91. hsa04062 Chemokine signaling pathway - Homo sapiens (human) (1) 92. hsa04012 ErbB signaling pathway - Homo sapiens (human) (1) 93. hsa05220 Chronic myeloid leukemia - Homo sapiens (human) (1) 94. hsa04540 Gap junction - Homo sapiens (human) (1) 95. hsa05210 Colorectal cancer - Homo sapiens (human) (1) |  |  |
| **IL-10** | 3586 | 1. hsa05145 Toxoplasmosis - Homo sapiens (human) (1)2. hsa05330 Allograft rejection - Homo sapiens (human) (1)3. hsa05150 Staphylococcus aureus infection - Homo sapiens (human) (1)4. hsa04672 Intestinal immune network for IgA production - Homo sapiens (human) (1)5. hsa05322 Systemic lupus erythematosus - Homo sapiens (human) (1)6. hsa04068 FoxO signaling pathway - Homo sapiens (human) (1)7. hsa05169 Epstein-Barr virus infection - Homo sapiens (human) (1)8. hsa04660 T cell receptor signaling pathway - Homo sapiens (human) (1)9. hsa05142 Chagas disease (American trypanosomiasis) - Homo sapiens (human) (1)10. hsa05146 Amoebiasis - Homo sapiens (human) (1)11. hsa05140 Leishmaniasis - Homo sapiens (human) (1)12. hsa04630 Jak-STAT signaling pathway - Homo sapiens (human) (1)13. hsa05133 Pertussis - Homo sapiens (human) (1)14. hsa04060 Cytokine-cytokine receptor interaction - Homo sapiens (human) (1)15. hsa05144 Malaria - Homo sapiens (human) (1)16. hsa05310 Asthma - Homo sapiens (human) (1)17. hsa05143 African trypanosomiasis - Homo sapiens (human) (1)18. hsa05321 Inflammatory bowel disease (IBD) - Homo sapiens (human) (1)19. hsa05152 Tuberculosis - Homo sapiens (human) (1)20. hsa05320 Autoimmune thyroid disease - Homo sapiens (human) (1) |  |  |
| **LIMK1 T508/** | 3984 | 1. hsa04360 Axon guidance - Homo sapiens (human) (1) 2. hsa04810 Regulation of actin cytoskeleton - Homo sapiens (human) (1) 3. hsa04666 Fc gamma R-mediated phagocytosis - Homo sapiens (human) (1) |  |  |
| **LIMK2 T505** | [3985](http://www.ncbi.nlm.nih.gov/entrez/query.fcgi?db=gene&cmd=Retrieve&dopt=full_report&list_uids=3985) | 1. hsa04666 Fc gamma R-mediated phagocytosis - Homo sapiens (human) (1) 2. hsa04810 Regulation of actin cytoskeleton - Homo sapiens (human) (1) 3. hsa04360 Axon guidance - Homo sapiens (human) (1) |  |  |
| Alpha-2-macroglobulin precursor | P01023 | calcium-dependent protein binding Source: AgBase enzyme binding Source: UniProtKB growth factor binding Source: UniProtKB GTPase activator activity Source: Reactome interleukin-1 binding Source: UniProtKB interleukin-8 binding Source: UniProtKB protease binding Source: BHF-UCL receptor binding Source: AgBase serine-type endopeptidase inhibitor activity Source: UniProtKB tumor necrosis factor binding Source: UniProtKB blood coagulation, intrinsic pathway Source: Reactome extracellular matrix disassembly Source: Reactome negative regulation of complement activation, lectin pathway Source: UniProtKB platelet degranulation Source: Reactome regulation of small GTPase mediated signal transduction Source: Reactome stem cell differentiation Source: Ensembl | R-HSA-114608. Platelet degranulation.   R-HSA-140837. Intrinsic Pathway of Fibrin Clot Formation.   R-HSA-1474228. Degradation of the extracellular matrix.   R-HSA-194223. HDL-mediated lipid transport.   R-HSA-194840. Rho GTPase cycle |  |
| Coagulation factor V precursor | P12259 | copper ion binding Source: InterPro serine-type endopeptidase activity Source: Ensembl blood circulation Source: Ensembl blood coagulation Source: Reactome COPII vesicle coating Source: Reactome ER to Golgi vesicle-mediated transport Source: Reactome platelet activation Source: InterPro platelet degranulation Source: Reactome response to vitamin K Source: Ensembl | R-HSA-114608. Platelet degranulation.  R-HSA-140875. Common Pathway of Fibrin Clot Formation.  R-HSA-204005. COPII (Coat Protein 2) Mediated Vesicle Transport.  R-HSA-5694530. Cargo concentration in the ER. |  |
| **Total** |  |  |  | **176** |

**Figure S4**: **Scatter plots of a. alpha-2 macroglobulin precursor vs. IL-10 (ρ=0.28, p=0.02) and ASK1 S83 (ρ=0.25, p=0.04)**

**
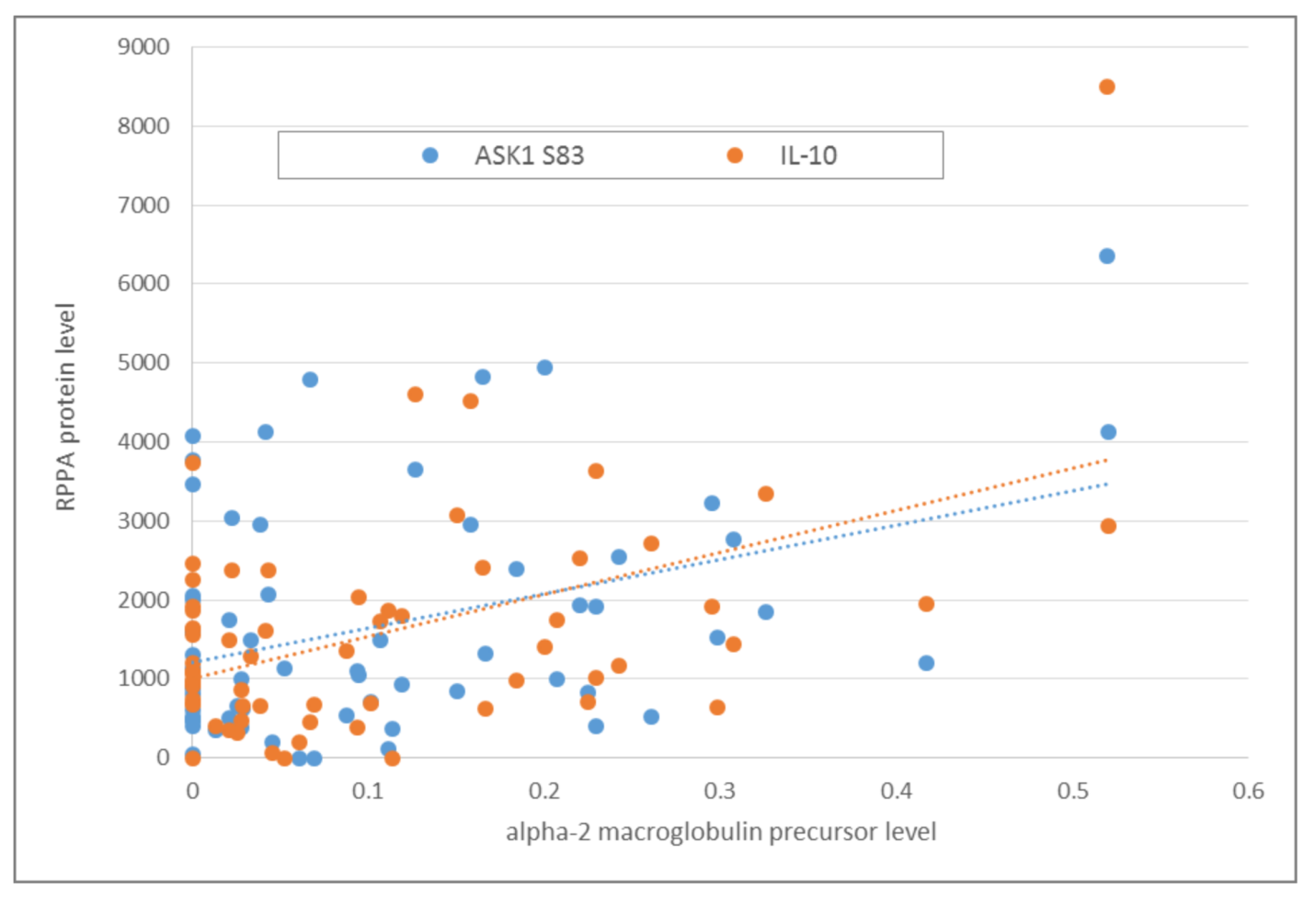
**
